# Long- and short-term effectiveness of traditional Chinese exercises in improving the overall physical capacity of patients with knee osteoarthritis: A systematic review and meta-analysis

**DOI:** 10.1097/MD.0000000000039520

**Published:** 2024-09-06

**Authors:** Boyuan Qiu, Weiwei Wang, Gangjian Tang, Sheng Chai, Xuan Zhang, Pengwei Zhou, Zhixue Ou

**Affiliations:** a Guangxi University of Traditional Chinese Medicine, Nanning, China; b The First Affiliated Hospital of Guangxi University of Traditional Chinese Medicine, Nanning, China; c Guilin Traditional Chinese Medicine Hospital, Guilin, China.

**Keywords:** baduanjin, knee osteoarthritis (KOA), Meta-Analysis, tai chi, traditional Chinese exercise, wuqinxi, yijinjing

## Abstract

**Background:**

The increasing global popularity of traditional Chinese exercise (TCE) provides substantial evidence of its significant efficacy in treating knee osteoarthritis (KOA). To assess the impact of different types of TCE and varying exercise durations on KOA patients, we conducted a systematic review and meta-analysis of randomized controlled trials (RCTs) on this topic.

**Methods:**

Two investigators extensively searched four electronic databases (PubMed, Embase, Cochrane, and Web of Science) from their inception until December 16, 2023, to identify all relevant RCTs on the use of TCE for KOA treatment. The included studies were assessed for risk of bias using the Cochrane Collaboration Risk of Bias Tool (CCRBT), and data analysis was performed using Stata 15.0.

**Results:**

A total of 20 RCTs, involving 1367 patients with KOA, met the inclusion criteria. Compared to the control group, TCE demonstrated significant improvement in three subscale scores of the Western Ontario and McMaster Universities Osteoarthritis Index (WOMAC) [Pain (SMD = −0.44; *P* = .0001); Stiffness (SMD = −0.35; *P* = .001); Physical function (SMD = −0.52; *P* = .0001)] and two subscale scores of the 36-item Short-Form (SF-36) [Physical score (WMD = 2.76; *P* = .001); Mental score (WMD = 2.49; *P* = .0001)] in KOA patients. Subgroup analysis showed that both long-term habitual exercise (over 12 weeks) and short-term exercise (within 12 weeks) were more effective than the control group in improving pain, joint stiffness, and physical function in KOA patients. Tai Chi, among the four TCE modalities analyzed, demonstrated improvements in all indicators.

**Conclusion:**

Based on the results of our meta-analysis, it can be concluded that both long-term and short-term TCE interventions are effective in alleviating the main symptoms of KOA and improving patients’ physical function. However, due to limited methodological quality and inconsistent outcome measures in the included RCTs, further high-quality RCTs with larger sample sizes and longer-term interventions are necessary to validate our findings before TCE can be recommended as a treatment for KOA.

## 1. Introduction

The pathogenesis of osteoarthritis (OA) involves various tissues within the joint, including the degeneration of joint,^[1]^ cartilage and meniscus due to collagen degradation, sclerosis and reconstruction of subchondral bone, and the presence of inflammatory pain in the infrapatellar fat pad-synovium anatomical functional unit caused by the expression of mechanicoreceptors and inflammatory factors.^[2–5]^ Studies focusing on knee and hip OA have shown that the global incidence of OA has increased by 9.3% from 1990 to 2017, with high rates in high-income areas like North America and among female populations.^[6]^ Among the different types of OA, knee osteoarthritis (KOA) is the most common, surpassing coxitis and cheirarthritis.^[7]^ KOA is associated with various factors including age, weight, and trauma,^[8,9]^ and it significantly impacts the quality of life due to its high rate of disability.^[10]^ Therefore, it is crucial to find effective therapies. Treatment options for KOA include conservative and surgical approaches. Surgical treatments range from total knee arthroplasty with implanted prostheses to minimally invasive procedures such as unicompartmental knee arthroplasty, osteotomy orthopedics, and arthroscopic surgery for joint debris removal or meniscus repairs.^[11]^ However, surgical therapies may come with problems such as postoperative pain, reduced quality of life, uncertain efficacy, and difficulties in bone union.^[12–14]^ As a result, conservative treatments for KOA have gained attention from medical professionals. Conservative therapy is the preferred treatment method according to the recommendations of the Osteoarthritis Research Society International.^[15]^ The latest clinical practice guidelines from the American Academy of Orthopaedic Surgeons suggest using topical medications, oral non-steroidal anti-inflammatory drugs (NSAIDs), and oral anesthetics for KOA.^[16]^ Intra-articular injections have also been proven to be more effective and safer than oral medications.^[17]^ However, long-term use of drug therapy can lead to adverse effects such as joint stiffness, gastrointestinal reactions, multi-organ toxicity, Kounis syndrome, and respiratory depression.^[17–20]^ On the other hand, cost-effective and safe physiotherapy, which has minimal side effects,^[21]^ has been highly recommended by the American Academy of Orthopaedic Surgeons, the American College of Rheumatology (ACR), and Osteoarthritis Research Society International for the treatment of KOA.^[15,16,22]^

Traditional Chinese exercise (TCE) refers to a series of ancient exercises originating from China that have been practiced for over 3000 years.^[23]^ This collection of exercises includes Qigong, Tai Chi, Baduanjin, Wuqinxi, and Yijinjing.^[24]^ Guided by the principles of the “Yin and Yang” doctrine and the holistic concept, TCE serves as a complementary therapy that aims to enhance blood circulation, regulate organ functions, and activate muscles and tendons through a combination of body movements, breath control, and meditation. By strengthening the body, preventing and treating diseases, and promoting overall well-being, TCE offers significant benefits for individuals seeking to improve their health.^[25,26]^ Multiple lines of evidence support the unique effectiveness of TCE in the treatment of various diseases. These include hypertension,^[27]^ myocytopenia,^[28]^ anxiety and drug addiction in individuals with substance abuse issues,^[29]^ metabolic diseases,^[30]^ cancer,^[31]^ chronic obstructive pulmonary disease,^[32]^ prevention of COVID-19, and enhancement of quality of life in patients recovering from COVID-19.^[33–35]^

TCE is currently being recognized as a promising therapy for musculoskeletal disorders.^[36]^ The evidence is mounting to support the notion that TCE can effectively alleviate the symptoms associated with KOA, such as pain, stiffness, impaired physical function, and compromised mental well-being.^[37–41]^ Given the inconsistent follow-up time and evaluation indicators, as well as the varying quality of the randomized controlled trials (RCTs) on the treatment of KOA with TCE, it is imperative to perform a comprehensive systematic review and meta-analysis of these studies. This will enable us to effectively assess and synthesize the available evidence. Furthermore, a comprehensive review and meta-analysis conducted previously demonstrated the significant efficacy of TCE in reducing stiffness symptoms and enhancing physical function in patients with KOA.^[42]^ It is worth noting, however, that the intervention techniques adopted by the experimental group in the existing literature solely comprised of Tai Chi and Baduanjin, neglecting other beneficial TCE practices like Yijinjing and Wuqinxi. Consequently, conducting further evidence-based research on the therapeutic effects of TCE in alleviating clinical symptoms among KOA patients would not only help broaden the treatment options available, but also enhance the overall management of this condition.

## 2. Methods

The evidence-based medicine guideline PRISMA^[43]^ was followed in reporting this systematic review and meta-analysis. The study was also registered on the PROSPERO website with the registration number CRD42023403655.

### 2.1. Search strategy

Using a search strategy that consisted of subject terms and free-text words, two independent investigators conducted a thorough search across four databases (PubMed, Embase, Cochrane, and Web of Science). The search encompassed literature published from the inception of these databases up until December 16, 2023. The primary focus of the search keywords revolved around knee osteoarthritis, knee joint, Qigong, Tai Chi, Yijinjing, Baduanjin, and Wuqinxi. Additionally, the investigators meticulously reviewed the references of the retrieved literature to ensure the inclusion of relevant sources that may have been missed. For more comprehensive search strategies, please refer to the Supplemental Digital Content 1, http://links.lww.com/MD/N485.

### 2.2. Inclusion and exclusion criteria

The inclusion criteria for this study on literature were as follows: Participants had to meet one or more of the diagnostic criteria for KOA as outlined by ACR, the American Rheumatism Association, the Kellgren-Lawrence scale, or have a clinical imaging basis or written evidence from physicians verifying the diagnosis of KOA.^[44–46]^ The trials had to investigate the efficacy of various Traditional Chinese Exercises (TCEs), including Qigong, Tai Chi, Baduanjin, Wuqinxi, or Yijinjing, by comparing them to a control group. The control group could consist of health education, waiting cohorts, or conventional physical therapy such as stretching exercises for the quadriceps. This comparison was done to assess the symptoms of patients with KOA. The outcome measurements included the Western Ontario and McMaster Universities Osteoarthritis Index (WOMAC), 36-item Short-Form (SF-36), the timed up and go test (TUG), Berg balance scale (BBS), and visual analogue scale (VAS).^[47–51]^ The study design specifically focused on RCTs.

The exclusion criteria for literature in this study were as follows: Reviews, meta-studies, study protocols, clinical guidelines, and conference abstracts were excluded. Participants with a history of knee trauma or surgery or rheumatoid arthritis were not included. Articles that were not published in English were excluded. Duplicate literature was not considered. The experimental group must have received TCE therapy alone as the intervention modality.

### 2.3. Study screening and data extraction

The retrieved literature was screened by two independent researchers according to the specified inclusion and exclusion criteria. Initially, the title and abstract of each piece of literature were read for screening purposes. Any studies that did not meet the inclusion criteria were then excluded. The remaining literature underwent a full text reading to determine its final inclusion. In instances where there was disagreement, a third researcher stepped in to facilitate a discussion and reach a final decision.

The two investigators independently conducted data extraction using a predetermined spreadsheet. The following information was extracted and recorded: General information: the first author’s name and year of publication. Study characteristics: the country where the study was conducted, basic characteristics of the subjects, sample size for each group, the intervention method used in each group, and the duration of follow-up. Outcome evaluation indicators.

### 2.4. Risk of bias assessment

Two researchers conducted an assessment of the risk of bias using Cochrane’s Risk of Bias RoB 2.0.^[52]^ This tool evaluates the risk of bias in 5 domains: randomization process, deviations from intended interventions, missing outcome data, outcome measurement, and selective outcome reporting. Each domain has 5 possible answers: yes, likely yes, likely no, no, and no information. Based on these responses, each domain will be classified into one of three risk levels: “low risk of bias,” “some concerns,” or “high risk of bias.” The 2 investigators cross-checked the results of the assessment. In case of disagreement, a third investigator would provide assistance in making the final decision.

### 2.5. Statistical analysis

Stata (version 17.0) (StataCorp, College Station) was employed for the purpose of conducting data analysis. Quantification of heterogeneity was achieved through the utilization of Cochran’s Q test and Higgins *I*^2^. Continuous variables with the same unit of measure were represented by WMD along with its corresponding 95% CI, whereas continuous variables with different units of measure were represented by SMD along with its corresponding 95% CI. In order to determine the presence of significant heterogeneity across studies, a result of *P* < .10 or *I*^2^ > 50% indicated so, leading to the adoption of a random-effects model. On the other hand, in cases where heterogeneity was not significant, a fixed-effects model was employed. Sensitivity analysis and subgroup analysis were carried out when heterogeneity was identified as excessively high in order to explore the sources of such heterogeneity. Funnel plots were utilized to provide a visual representation of publication bias, while the statistical testing of publication bias was conducted using the Egger test. A *P* > .05 suggested the presence of publication bias, and further processing was performed using the trim-and-fill method. Additionally, additional sensitivity analysis tests were carried out in order to assess the stability of the study results. A *P* < .05 indicated that the pooled statistics of the included studies were statistically significant.

### 2.6. Ethics approval

All analyses were based on previous published studies, all analyses were conducted, therefore, ethical approval and patient consent are not necessary for this study.

## 3. Results

### 3.1. Study screening

**Figure 1.**
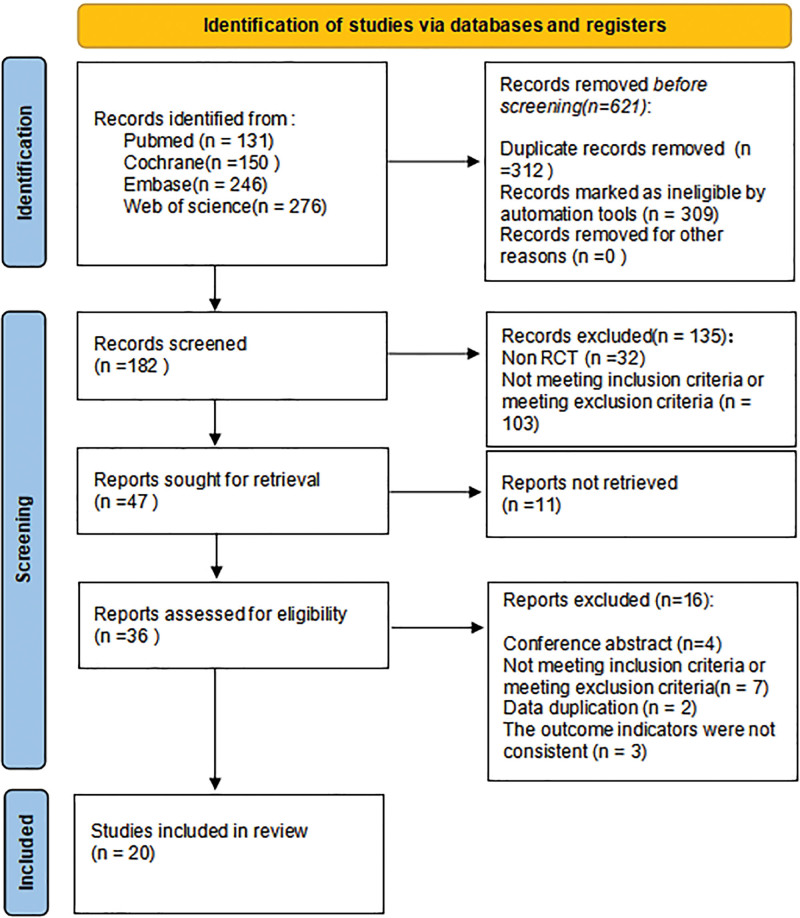
Flowchart of study selection.

Figure 1 illustrates that a total of 803 articles were obtained, out of which 612 were excluded either because they were identified as duplicates by endnote or because their titles were clearly unrelated to the study objective. Additionally, 135 articles that did not meet the eligibility criteria were excluded after reviewing their abstracts. The remaining 47 articles underwent a thorough reading, resulting in the exclusion of 27 articles based on the predefined inclusion and exclusion criteria. Ultimately, this meta-analysis included a total of 20 RCTs.

### 3.2. Characteristics of the included studies and subjects

The 20 studies included in the analysis involved a total of 1367 patients with KOA. These studies were published in English between 2000 and 2022,^[37–41,53–67]^ and were conducted in China, the United States, South Korea, and Iran. The patients in these studies either met one or more diagnostic criteria for KOA, such as those set by the ACR, American Rheumatism Association, Kellgren and Lawrence (KL) scale, or had a clinical imaging basis or written evidence from physicians confirming the diagnosis of KOA.

The included studies involved a total of 20 different research articles. The age range of the participants in these studies ranged from 52 to 78 years old. Out of the 20 studies, 8 exclusively focused on female participants,^[38,40,56,58–61,65]^ while 11 studies included both male and female participants.^[37,39,41,53–55,62–64,66,67]^ Only one study exclusively enrolled male participants.^[57]^ In terms of the intervention methods, the majority of the studies utilized Tai Chi, while 2 studies used Baduanjin and Wuqinxi respectively,^[38,39,41,56]^ and only 1 study used Yijinjing.^[55]^ The follow-up periods for these studies ranged from 8 to 24 weeks. Table 1 provides more detailed information about the specific characteristics of the included studies.

**Table 1:** Characteristics of included studies.

References	Research location	Participant characteristics			Pre-baseline exercise/physical therapy status, n (%)		Intervention plan			Measurement results
		Sample size (male/female)	Mean age or age range	Diagnostic criteria for KOA	Experimental group	Control group	Experimental group	Control group	Intervention time	
^[38]^	China	IG: 132 (0/132) CG: 134(0/134)	IG: 71 ± 2.92 CG: 69 ± 3.72	ACR	Physical therapy, 49 (37.1%)	Physical therapy, 58 (43.3%)	Wuqinxi 6 × 60 min/wk	None	24 wk	WOMAC SF-36
^[53]^	USA	IG: 22 (3/19) CG: 19 (4/15)	IG: 70.89 ± 9.8 CG: 68.89 ± 8.9	ARA	Exercise, 14 (63.6%)	Exercise, 14 (73.7%)	Tai chi pre-12 weeks 3 × 40 min/wk post-6 weeks none	Team meeting pre-6 weeks 3 × 40 min/wk post-12 weeks none	18 wk	WOMAC VAS
^[62]^	USA	IG: 20 (4/16) CG: 20(14/6)	IG: 63 ± 8.1 CG: 68 ± 7.0	ACR	F1	F1	Tai chi pre-12 weeks 2 × 60 min/wk in classroom 7 × 20 min/wk at home post-36 weeks exercise Tai chi consciously at home	Team meeting pre-12 weeks 2 × 60 min/wk post-36 weeks none	48 wk	WOMAC VAS SF-36
^[54]^	korea	IG: 29 (2/27) CG: 15(1/14)	IG: 70.2 ± 4.8 CG: 66.9 ± 6.0	KL scale	F1	F1	Tai chi 2 × 60 min/wk	None	8 wk	WOMAC SF-36
^[39]^	China	IG: 49 (17/32) CG: 49(20/29)	IG: 70.7 ± 9.36 CG: 70.2 ± 10.35	KL scale or clinical symptoms	F1	F1	Wuqinxi 4 × 60 min/wk	Lower extremity training 4 × 60 min/wk	24 wk	WOMAC BBS TUG
^[55]^	China	IG: 25 (4/21) CG: 25 (9/16)	IG: 55.76 ± 8.37 CG: 53.40 ± 10.66	Radiographic evidence and clinical symptoms	F1	F1	yijinjing 2 × 60 min/wk	Quadriceps training 2 × 60 min/wk	12 wk	WOMAC SF-36 BBS VAS
^[56]^	China	IG: 14 (0/14) CG: 14 (0/14)	IG: 65.4 ± 8.2 CG: 64.6 ± 6.7	Radiographic evidence	F1	F1	baduanjin pre- 8 weeks 5 × 30 min/wk post- 16 weeks none	None	24 wk	WOMAC SF-36
^[57]^	Iran	IG: 12 (12/0) CG: 12 (12/0)	IG: 51.6 ± 08.69 CG: 54.6 ± 92.27	Radiographic evidence	F1	F1	Tai chi 3 × 60 min/wk	Quadriceps training 3 × 60 min/wk	8 wk	BBS VAS
^[58]^	China	IG: 52 (0/52) CG: 40 (0/40)	IG: 66.32 ± 4.16 CG: 66.32 ± 4.16	Radiographic evidence and clinical symptoms	F1	F1	Tai chi 3 × 60 min/wk	Team meeting 3 × 60 min/wk	24 wk	WOMAC VAS
^[59]^	China	IG: 23 (0/23) CG: 23 (0/23)	IG: 64.61 ± 3.40 CG: 64.53 ± 3.43	ARA	F1	F1	Tai chi 3 × 60 min/wk	Team meeting 60 min/wk	24 wk	SF-36 BBS TUG
^[60]^	China	IG: 23 (0/23) CG: 23 (0/23)	IG: 64.61 ± 3.40 CG: 64.53 ± 3.43	ARA	F1	F1	Tai chi 3 × 60 min/wk	Team meeting 60 min/wk	24 wk	WOMAC
^[40]^	China	IG: 20 (0/20) CG: 20 (0/20)	IG: 64.15 ± 8.56 CG: 64.15 ± 8.56	Radiographic evidence	F1	F1	Tai chi 3 × 60 min/wk	Team meeting 60 min/wk	12 wk	WOMAC SF-36 BBS TUG
^[61]^	China	IG: 18 (0/18) CG: 17 (0/17)	IG: 62.89 ± 2.79 CG: 63.47 ± 2.85	ARA	F1	F1	Tai chi the first 8 weeks 2 × 45 min/wk the second 8 weeks 3 × 45 min/wk the third 8 weeks 3 × 45 min/wk	Stretching 45 min/wk	24 wk	WOMAC
^[37]^	China	IG: 36(4/32) CG: 32 (2/30)	IG: 77.4 ± 5.9 CG: 75.4 ± 6.4	ACR	F2	F2	Tai chi 2 × 60 min/wk	Team meeting 2 × 60 min/wk	12 wk	TUG
^[41]^	China	IG 41 (7/34) CG: 43(8/35)	IG: 64.74 ± 2.80 CG: 65.70 ± 3.50	clinical symptoms	F1	F1	baduanjin 3 × 40 min/wk	Quadriceps training 3 × 40 min/wk	24 wk	WOMAC SF-36
^[63]^	USA	IG: 12 (9/3) CG: 6(4/2)	IG: 68.1 ± 5.3 CG: 70.5 ± 5.0	ACR	F1	F1	Tai chi 2 × 60 min/wk	None	10 wk	WOMAC 6-MWT TUG
^[64]^	Iran	IG: 16 CG: 16	IG: 55.25 ± 5.72 CG: 56.06 ± 6.13	KL scale	F1	F1	Tai chi 3 × 20 min/wk	None	8 wk	TUG
^[65]^	korea	IG: 22 (0/22) CG: 21(0/21)	IG: 64.8 ± 6.0 CG: 62.5 ± 5.6	Radiographic evidence and clinical symptoms	Exercise, 16 (72.7%)	Exercise, 15 (71.4%)	Tai chi pre- 2 weeks 3 × 60 min/wk post- 10 weeks 4 × 60 min/wk	None	12 wk	WOMAC
^[66]^	USA	IG: 28 (6/22) CG: 27(9/18)	IG: 78.89 ± 6.91 CG: 78.93 ± 8.30	clinical symptoms	F1	F1	Tai chi 3 × 60min/wk Participate in health culture classes	Participate in health culture classes	21 wk	WOMAC
^[67]^	USA	IG: 106 (31/75) CG: 98(30/68)	IG: 60.3 ± 10.5 CG: 60.1 ± 10.5	ACR or Radiographic evidence	F1	F1	Tai chi 2 × 60min/wk	Physicotherapy pre- 6 weeks 2 × 60 min/wk in classroom post- 6 weeks exercise consciously at home	52 wk	WOMAC SF-36

F1: The inclusion criteria for the study include conditions such as no exercise or not receiving physical therapy, or the exclusion criteria include conditions such as habitual exercise or receiving physical therapy, meaning that participants in the study did not exercise or receive physical therapy before baseline.

F2: The information not mentioned in the article.

ACR = American College of Rheumatology, ARA = American Rheumatism Association, BBS = Berg balance scale, KL scale = Kellgren-Lawrence scale, KOA = knee osteoarthritis, SF-36 = 36-item short-form, TUG = timed up and go test, VAS = visual analogue scale, WOMAC = Western Ontario and McMaster Universities Osteoarthritis Index.

### 3.3. Risk of bias assessment

**Figure 2.**
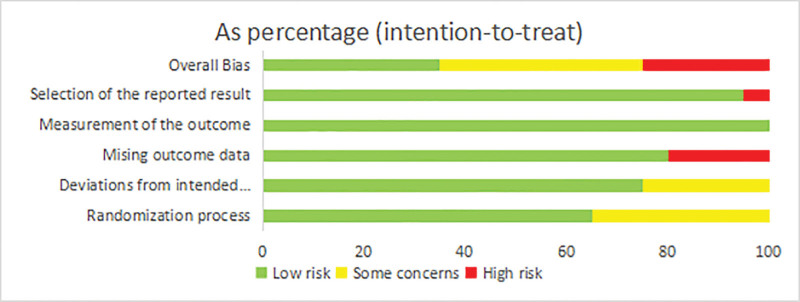
Analysis of the risk of bias for the included trials in this study.

**Figure 3.**
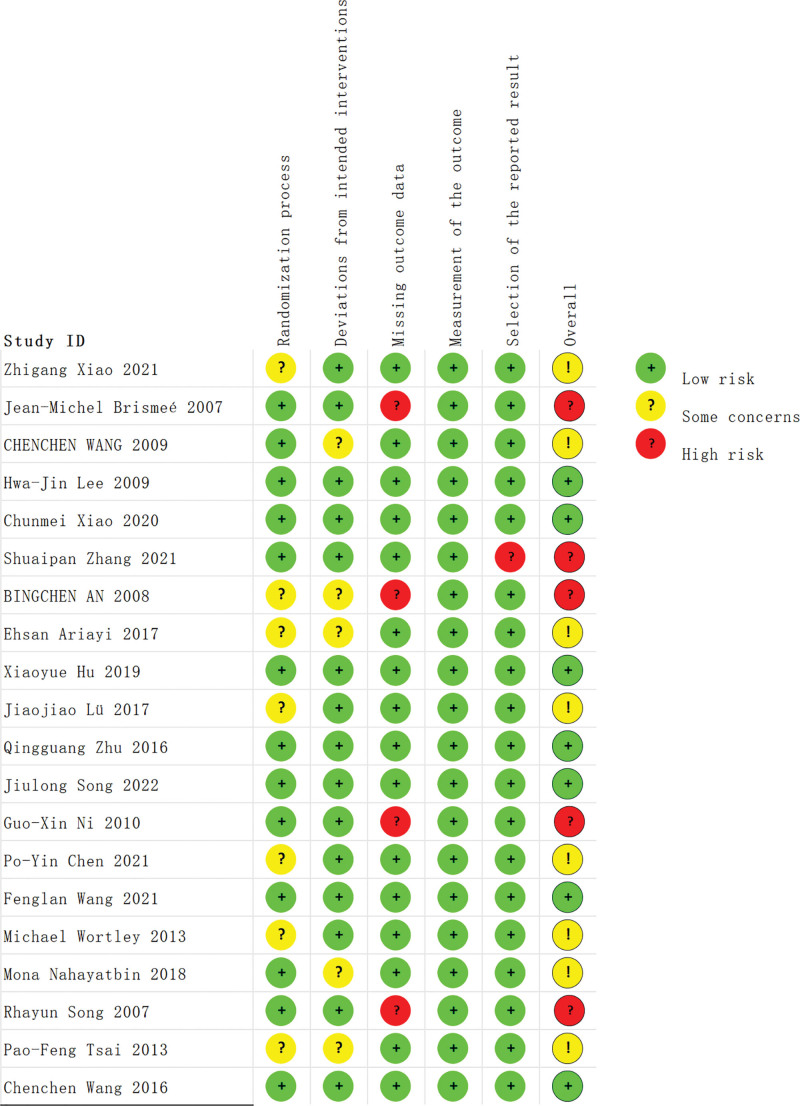
Summary of the risk of bias for the included trials in this study.

The risk of bias assessment results are presented in Figures 2 and 3. In total, 35% of the studies were deemed to have a low risk of bias, while 40% were found to have some concerns, and 25% were assessed as high risk of bias. Among these studies, 7 were identified as having some concerns regarding the lack of specific reporting of the random sequence generation.^[37,38,56,57,59,63,66]^ Additionally, 5 studies^[56,57,62,64,66]^ were assessed as having some concerns due to insufficient reporting of allocation concealment methods. Furthermore, 4 studies^[53,56,61,65]^ were determined to have a high risk of bias due to missing data caused by a significant number of participants lost to follow-up during the study process. Lastly, 1 study^[55]^ was evaluated as having a high risk of bias in terms of selective reporting, which stemmed from inconsistencies between the final reported measurement results and the methods section. It should be noted that the risk of measurement bias was found to be low for all included studies.

### 3.4. Primary outcomes

#### 3.4.1. WOMAC

##### 3.4.1.1. Meta-analysis

A total of 16 studies have reported the measurement results of the WOMAC scale. The scale, consisting of 24 items, is a self-administered questionnaire that is divided into three subscales: pain, stiffness, and physical function. The measurement results were reported in studies numbered.^[38–41,53–56,58,60–63,65–67]^ The primary focus of the current study was to analyze the improvement of TCE on KOA using the measurement results from each of the three subscales mentioned above. However, it should be noted that one study could not be included in the WOMAC outcome analysis due to the unavailability of outcome data. This study is referenced as study number.^[40]^

**Figure 4.**
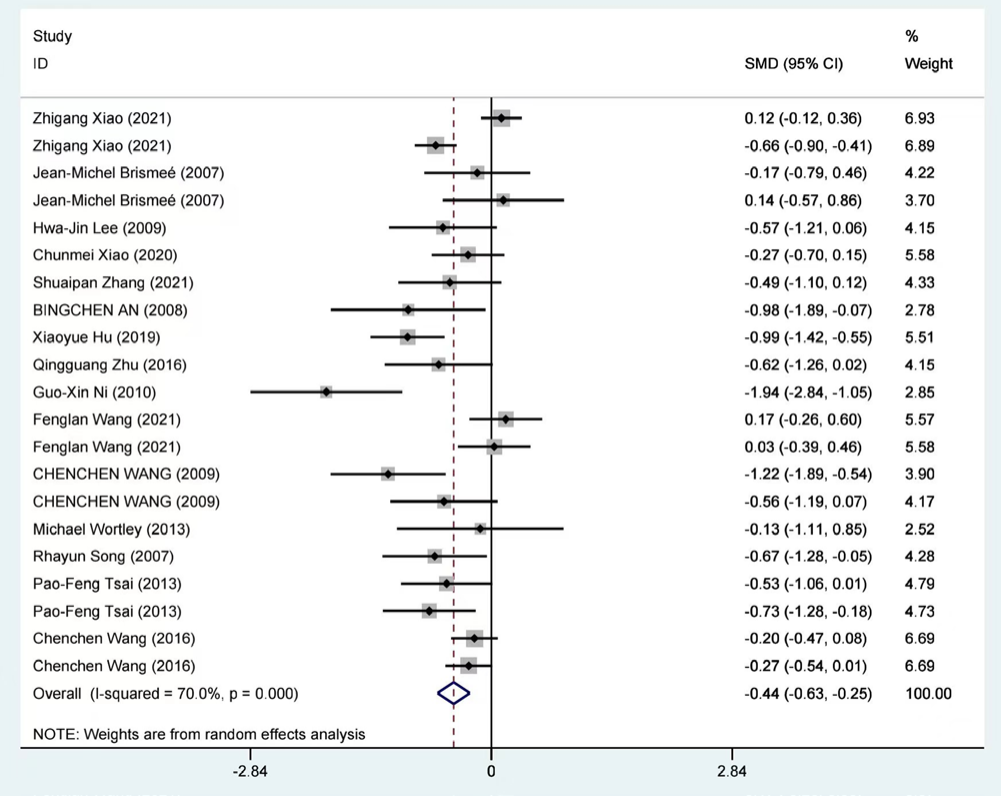
Meta-analysis of the TCE group compared with the control group on WOMAC pain score. TCE = traditional Chinese exercise, WOMAC = Western Ontario and McMaster Universities Osteoarthritis Index.

A meta-analysis was conducted on the results of 15 studies involving 1144 patients, reporting the WOMAC pain subscale scores. The studies, which were meta-analyzed.^[38,39,41,53–56,58,60–63,65–67]^ Due to the significant statistical heterogeneity observed among the results of the studies (*I*^2^ = 70.0%), a random-effects model was employed for the meta-analysis. The findings of the studies indicated that TCE (The Treatment of Choice) exhibited greater efficacy in improving WOMAC pain scores compared to the control group. The standardized mean difference (SMD) was −0.44 (95% CI: −0.63 to −0.25, *P* = .0001). The results of the meta-analysis of WOMAC pain scores are depicted in Figure 4.

**Figure 5.**
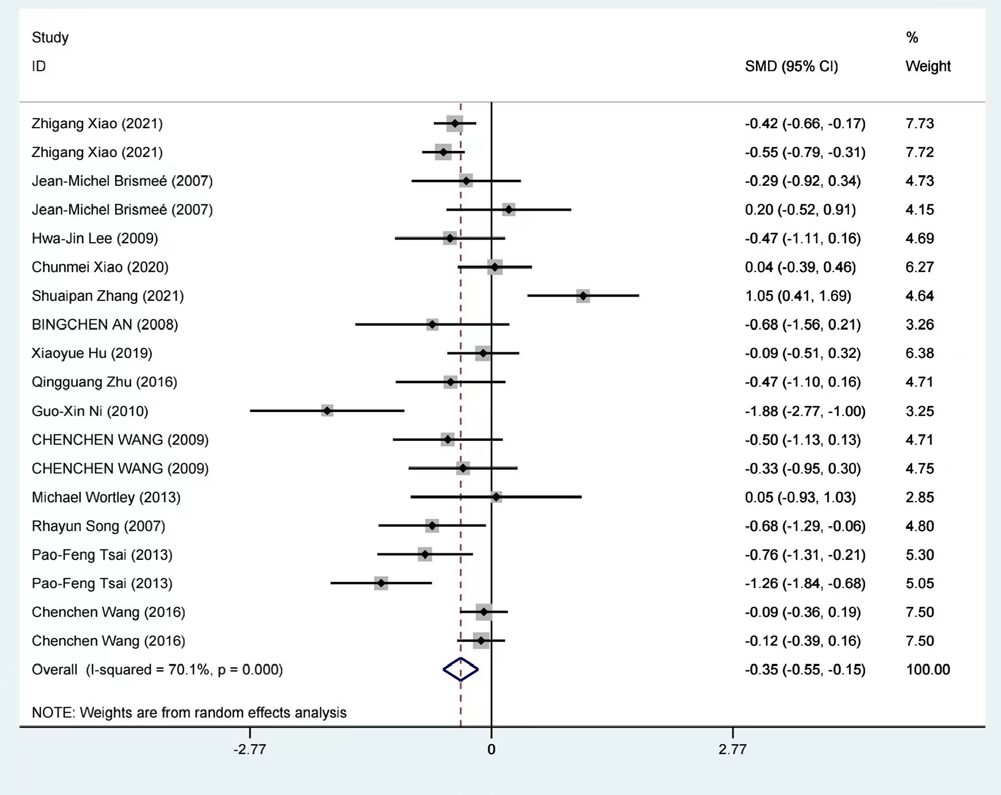
Meta-analysis of the TCE group compared with the control group on WOMAC stiffness score. TCE = traditional Chinese exercise, WOMAC = Western Ontario and McMaster Universities Osteoarthritis Index.

A meta-analysis was conducted on the results of 14 studies involving a total of 1060 patients^[38,39,53–56,58,60–63,65–67]^ to evaluate the efficacy of TCE in improving WOMAC stiffness scores compared to a control group. Due to significant statistical heterogeneity between the study results (*I*^2^ = 70.1% 25), a random-effects model was applied for the meta-analysis. The findings revealed a significant improvement in WOMAC stiffness scores with TCE compared to the control group (SMD = −0.35; 95% CI = −0.55 to −0.15; *P* = .001). This meta-analysis is visually represented in Figure 5.

**Figure 6.**
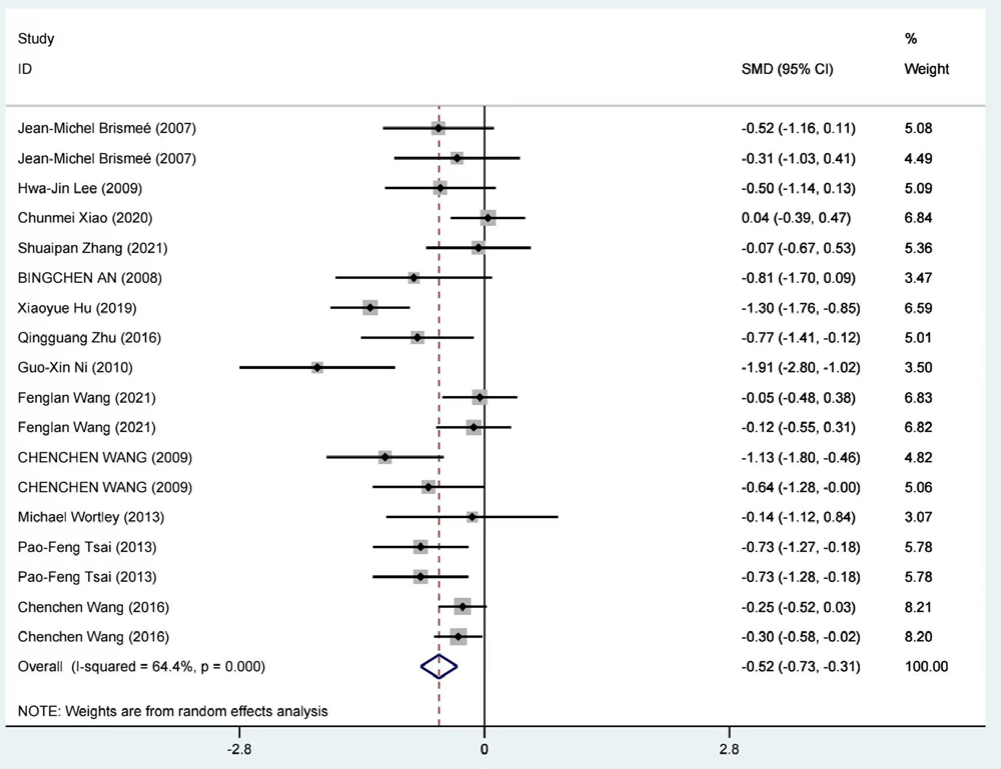
Meta-analysis of the TCE group compared with the control group on the WOMAC physical function score. TCE = traditional Chinese exercise, WOMAC = Western Ontario and McMaster Universities Osteoarthritis Index.

Meta-analysis was conducted on the WOMAC physical function subscale results from 14 studies involving 1101 patients.^[38,39,41,53–56,58,60–63,66,67]^ Due to significant statistical heterogeneity among the study results (*I*^2^ = 64.4% 25), a random-effects model was used for the meta-analysis. The findings indicated that TCE showed greater effectiveness in improving WOMAC physical function scores compared to the control group (SMD = −0.52; 95%CI = −0.73 to −0.31; *P* = .0001). The meta-analysis results for the WOMAC physical function score are presented in Figure 6.

##### 3.4.1.2. Subgroup analysis

We conducted subgroup analysis to evaluate the efficacy of TCE on KOA, based on the different durations of exercise and types of physical activity. Moreover, considering the substantial heterogeneity observed in the WOMAC outcomes, subgroup analysis can be employed to examine the possible factors contributing to this heterogeneity. The subgroup analysis results of WOMAC are presented in Tables 2 and 3; Supplemental Digital Content 2 and 3, http://links.lww.com/MD/N485.

**Table 2:** Results of WOMAC subgroup analysis (length of follow-up).

Measurements	Number of included studies	Subgroup	*I*^2^ value (%)	SMD (95% CI)	*P* value
WOMAC (pain)	11	≤12 wk	62.7	**−0.35 (−0.60, −0.10**)	.007
	10	˃12 wk	70.1	**−0.53 (−0.79, −0.26**)	.001
WOMAC (stiffness)	10	≤12 wk	65.9	**−0.28 (−0.57, 0.00**)	.048
	9	˃12 wk	76.0	**−0.43 (−0.74, −0.12**)	.007
WOMAC (function)	9	≤12 wk	33.3	**−0.41 (−0.64, −0.18**)	.0001
	9	˃12 wk	76.9	**−0.61 (−0.96, −0.26**)	.001

Bold values in the tables indicate meaningful results with *P* < .05.

WOMAC = Western Ontario and McMaster Universities Osteoarthritis Index.

**Table 3:** Results of WOMAC subgroup analysis (type of exercise).

Measurements	Number of included studies	Subgroup	*I*^2^ value (%)	SMD (95% CI)	*P* value
WOMAC (pain)	2	Wuqinxi	89.7	−0.27 (−0.79, 0.25)	.313
	10	Tai Chi	59.6	**−0.56 (−0.79, −0.34**)	.0001
	1	Yijinjing	None	−0.49 (−1.10, 0.12)	.114
	2	Baduanjin	60.5	−0.11 (−0.61, 0.39)	.664
WOMAC (stiffness)	2	Wuqinxi	63.7	**−0.36 (−0.64, −0.08**)	.012
	10	Tai Chi	63.1	**−0.43 (−0.67, −0.20**)	.0001
	1	Yijinjing	None	1.05 (0.41, 1.69)	.001
	1	Baduanjin	None	−0.68 (−1.56, 0.21)	.132
WOMAC (function)	1	Wuqinxi	None	0.04 (−0.39, 0.47)	.855
	9	Tai Chi	63.3	**−0.68 (−0.92, −0.43**)	.0001
	1	Yijinjing	None	−0.07 (−0.67, 0.53)	.822
	2	Baduanjin	12.4	−0.17 (−0.49, 0.14)	.282

Bold values in the tables indicate meaningful results with *P* < .05.

WOMAC = Western Ontario and McMaster Universities Osteoarthritis Index.

The results of subgroup analysis demonstrated that both long-term (>12 weeks) and short-term (≤12 weeks) therapeutic exercise interventions yielded significant improvements in pain, stiffness, and physical function scores as assessed by the WOMAC in comparison to the control group. Specifically, Tai Chi exhibited significant improvements in pain, stiffness, and physical function scores, while Wuqinxi demonstrated significant improvements only in stiffness scores. Moreover, the heterogeneity observed in the meta-analysis results of the WOMAC physical function subscale appears to be influenced by the duration of follow-up and the type of exercise utilized, whereas the heterogeneity in the pain and stiffness subscales should not be attributed to these factors.

#### 3.4.2. SF-36

##### 3.4.2.1. Meta-analysis

A total of 8 studies have reported SF-36 measurements.^[38,40,41,54,56,59,62,67]^ The SF-36 is a comprehensive questionnaire consisting of 36 items, designed to assess both physical functioning and psychological well-being.^[36]^ The study excluded in the analysis did not provide information on whether the score was utilized to assess physical function or psychological status27.

**Figure 7.**
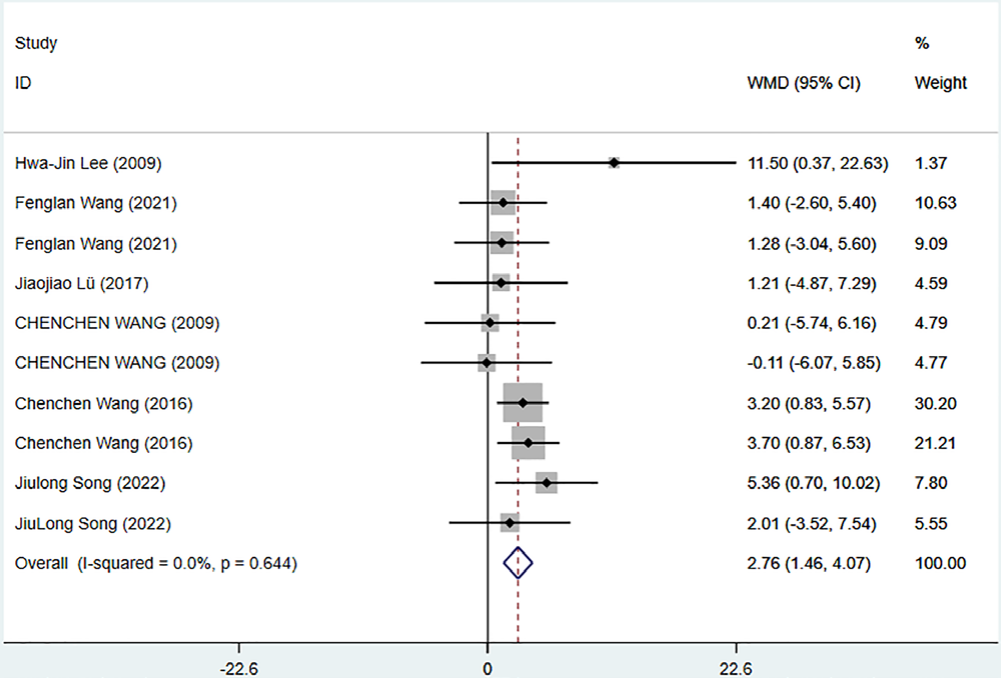
Meta-analysis of the TCE group compared with the control group on SF-36 physical function score. TCE = traditional Chinese exercise.

A meta-analysis was conducted based on 7 studies involving 486 patients^[40,41,54,56,59,62,67]^ to evaluate the results of the SF-36 physical function score. In order to account for the minimal statistical heterogeneity across the studies (*I*^2^ = 0%), a fixed-effects model was utilized. The findings indicated that TCE displayed superior effectiveness in enhancing SF-36 physical function scores compared to the control group (WMD = 2.76; 95% CI = 1.46–4.07; *P* = .001). Figure 7 illustrates the meta-analysis outcomes of the SF-36 physical function score.

**Figure 8.**
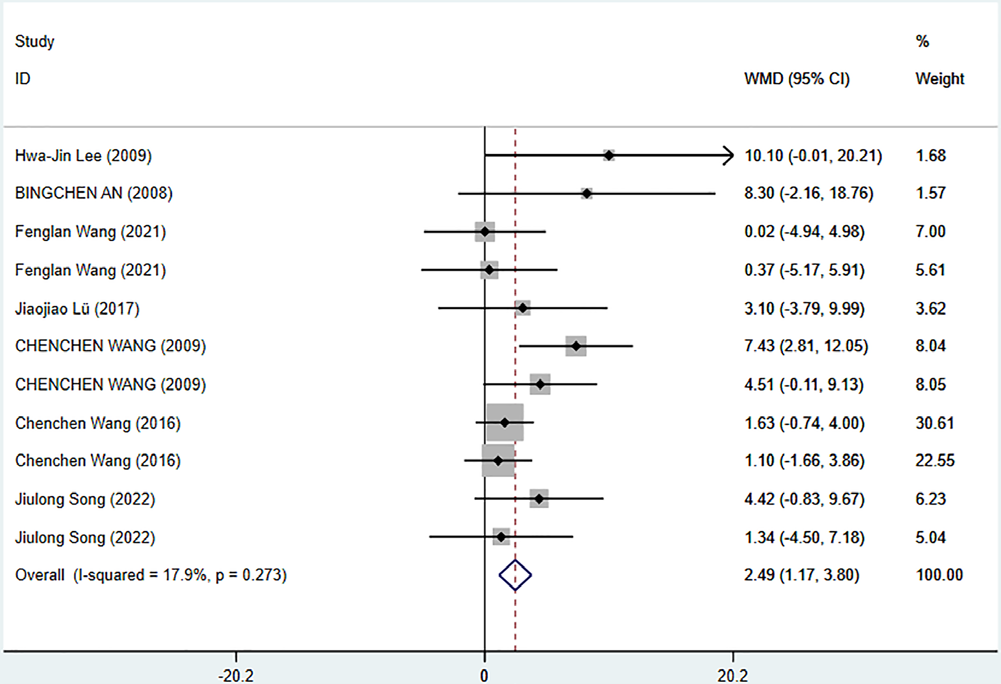
Meta-analysis of the TCE group compared with the control group on the SF-36 psychological status score. TCE = traditional Chinese exercise.

Meta-analysis was conducted on the psychological status scores measured by SF-36 in 7 studies that involved 486 patients.^[40,41,54,56,59,62,67]^ A fixed-effects model was used for the meta-analysis due to the low statistical heterogeneity among the results of the studies (*I*^2^ = 17.9% 25). The findings demonstrated that TCE significantly improved SF-36 mental health scores compared to the control group (WMD = 2.49; 95% CI = 1.17–3.80; *P* = .0001). The meta-analysis results for SF-36 psychological status scores are presented in Figure 8.

##### 3.4.2.2. Subgroup analysis

Further analysis was conducted to examine the effects of different durations and modalities of TCE in treating KOA. The findings revealed significant improvements in the physical function scores of SF-36 with both long-term (>12 weeks) and short-term TCE (≤12 weeks), compared to the control group. Specifically, short-term TCE (≤12 weeks) demonstrated effectiveness in enhancing the SF-36 mental health scores. Additionally, Tai Chi was found to be a beneficial intervention for improving both physical function and mental health scores of SF-36. Detailed results can be found in Tables 4 and 5, as well as the Supplemental Digital Content 4 and 5, http://links.lww.com/MD/N485.

**Table 4:** Results of SF-36 subgroup analysis (length of follow-up).

Measurements	Number of included studies	Subgroup	*I*^2^ value (%)	WMD (95% CI)	*P* value
SF-36 (physical)	5	≤12 wk	14.8	**3.10 (1.34, 4.86**)	.001
	5	˃12 wk	0	**2.35 (0.41, 4.29**)	.017
SF-36 (mental)	6	≤12 wk	46.6	**3.04 (1.27, 4.80**)	.001
	5	˃12 wk	0	1.81 (−0.15, 3.77)	.07

Bold values in the tables indicate meaningful results with *P* < .05.

SF-36 = 36-item short-form.

**Table 5:** Results of the SF-36 subgroup analysis (type of exercise).

Measurements	Number of included studies	Subgroup	*I*^2^ value (%)	WMD (95% CI)	*P* value
SF-36 (physical)	5	Tai Chi	0	**3.11 (1.66, 4.57**)	.001
	1	Baduanjin	0	1.34 (−1.59, 4.28)	.369
SF-36 (mental)	5	Tai Chi	25.3	**2.72 (1.30, 4.13**)	.0001
	2	Baduanjin	3.4	1.08 (−2.41, 4.56)	.545

Bold values in the tables indicate meaningful results with *P* < 0.05.

SF-36 = 36-item short-form.

#### 3.4.3. Secondary outcomes

##### 3.4.3.1. Meta-analysis

The secondary outcomes of the study included TUG test, BBS, and VAS. The results of the analysis showed that the intervention group, known as TCE, performed better than the control group in terms of improving BBS and TUG scores. However, no significant difference was observed between the 2 groups in terms of improving VAS scores. For further information, please refer to Table 6 and the Supplemental Digital Content 6, http://links.lww.com/MD/N485.

**Table 6:** Results of meta-analysis of the TCE group compared with the control group on TUG, BBS, and VAS scores.

Measurements	Number of included articles	Number of patients involved	*I*^2^ value (%)	WMD (95% CI)	*P* value
TUG	5	270	85.6	**−0.73 (−1.37, −0.09**)	.025
BBS	5	258	0	**1.22 (0.49, 1.95**)	.001
VAS	5	247	79.3	−0.41 (−1.65, 0.83)	.517

Bold values in the tables indicate meaningful results with *P* < .05.

BBS = Berg balance scale, TCE = traditional Chinese exercise, TUG = timed up and go test, VAS = visual analogue scale.

##### 3.4.3.2. Subgroup analysis

To further investigate the variations in the effectiveness of different durations and types of TCE in treating KOA, we conducted a subgroup analysis. The purpose of this analysis was 2-fold: firstly, to identify the sources of heterogeneity in the results of TUG and VAS; and secondly, to examine the impact of exercise durations on the outcomes. The findings indicated that long-term TCE (more than 12 weeks) was significantly more effective than the control group in improving BBS scores. Notably, Tai Chi demonstrated significant improvements in TUG, VAS, and BBS scores. Interestingly, the subgroup analysis based on the *I*^2^ index revealed that exercise duration played a crucial role in the observed heterogeneity in the meta-analysis results of TUG and VAS. For a detailed breakdown of the results, please refer to Tables 7 and 8, and the Supplemental Digital Content 7 and 8, http://links.lww.com/MD/N485.

**Table 7:** Results of subgroup analysis of TUG, VAS, and BBS (length of follow-up).

Measurements	Number of included articles	Subgroup	*I*^2^ value (%)	WMD (95% CI)	*P* value
TUG	3	≤12 wk	93.4	−1.39 (−3.52, 0.73)	.199
	3	˃12 wk	35.7	−0.37 (−0.78, 0.05)	.082
VAS	3	≤12 wk	66.3	0.06 (−1.49, 1.62)	.935
	2	˃12 wk	43.9	−1.02 (−2.21, 0.17)	.094
BBS	3	≤12 wk	0	0.95 (−0.20, 2.11)	.104
	3	˃12 wk	26.6	**1.40 (0.45, 2.34**)	.004

Bold values in the tables indicate meaningful results with *P* < 0.05.

BBS = Berg balance scale, TUG = timed up and go test, VAS = visual analogue scale.

**Table 8:** Results of subgroup analysis of TUG, VAS, and BBS (type of exercise).

Measurements	Number of included articles	Subgroup	*I*^2^ value (%)	WMD (95% CI)	*P* value
TUG	1	Wuqinxi	None	0.50 (−0.59, 1.59)	.369
	4	Tai Chi	87.2	**−0.93 (−1.62, −0.25**)	.008
VAS	3	Tai Chi	0	**−1.07 (−1.78, −0.37**)	.003
	1	Yijinjing	None	0.72 (0.22, 1.22)	.005
BBS	1	Wuqinxi	None	2.70 (0.14, 5.26)	.038
	1	Yijinjing	None	0.56 (−1.20, 2.32)	.532
	3	Tai Chi	0	**1.21 (0.37, 2.05**)	.005

Bold values in the tables indicate meaningful results with *P* < 0.05.

BBS = Berg balance scale, TUG = timed up and go test, VAS = visual analogue scale.

### 3.5. Sensitivity analysis

We conducted sensitivity analyses on the WOMAC, SF-36, TUG, BBS, and VAS results from the 20 studies included in order to assess the robustness of the combined findings. The findings revealed that the studies’ data points fell within the original confidence interval’s effect size, indicating the stability of the analysis results. Supplementary Digital Content 9, http://links.lww.com/MD/N485 provides detailed results of the sensitivity analysis.

### 3.6. Assessment of publication bias

**Figure 9.**
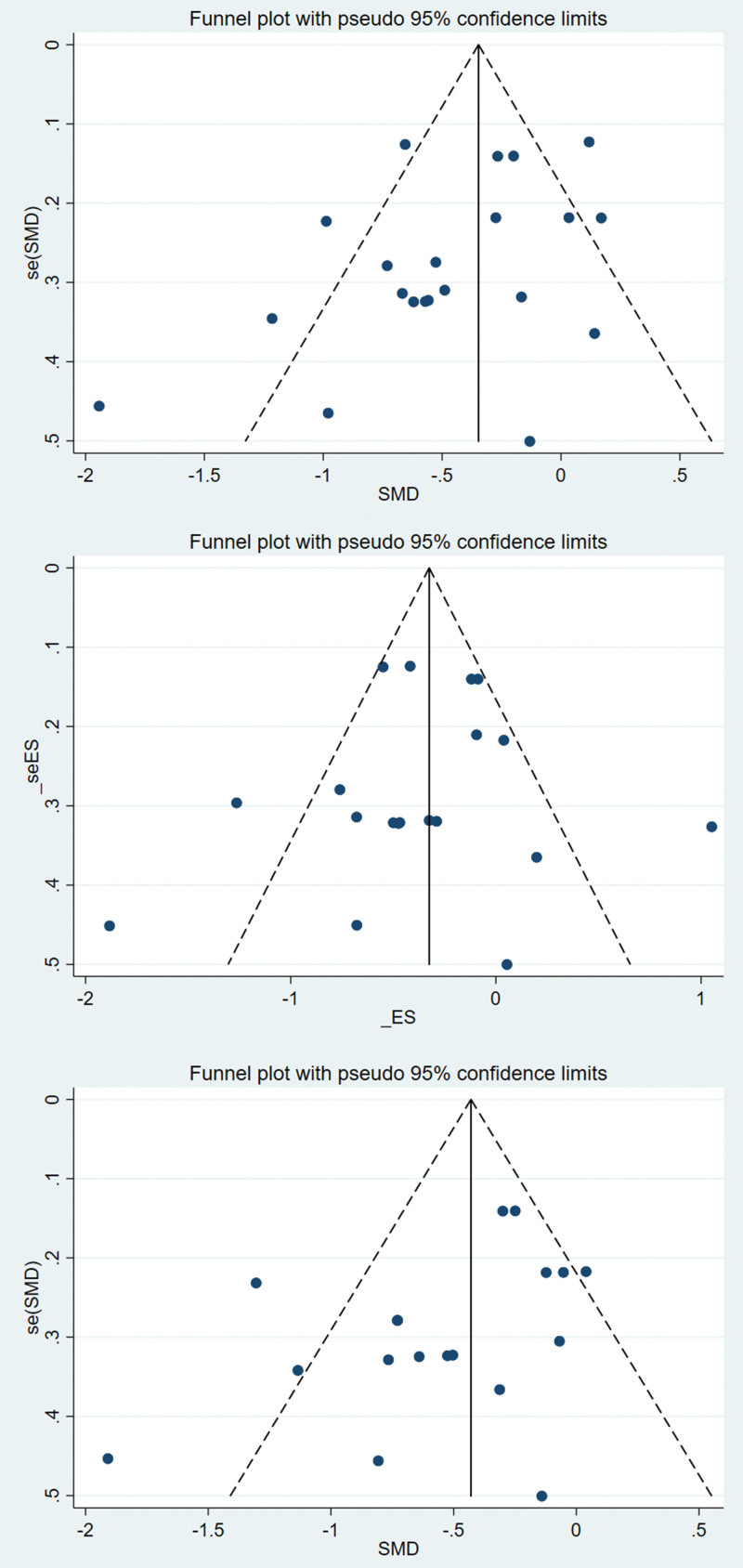
Funnel plot of WOMAC. WOMAC = Western Ontario and McMaster Universities Osteoarthritis Index.

**Figure 10.**
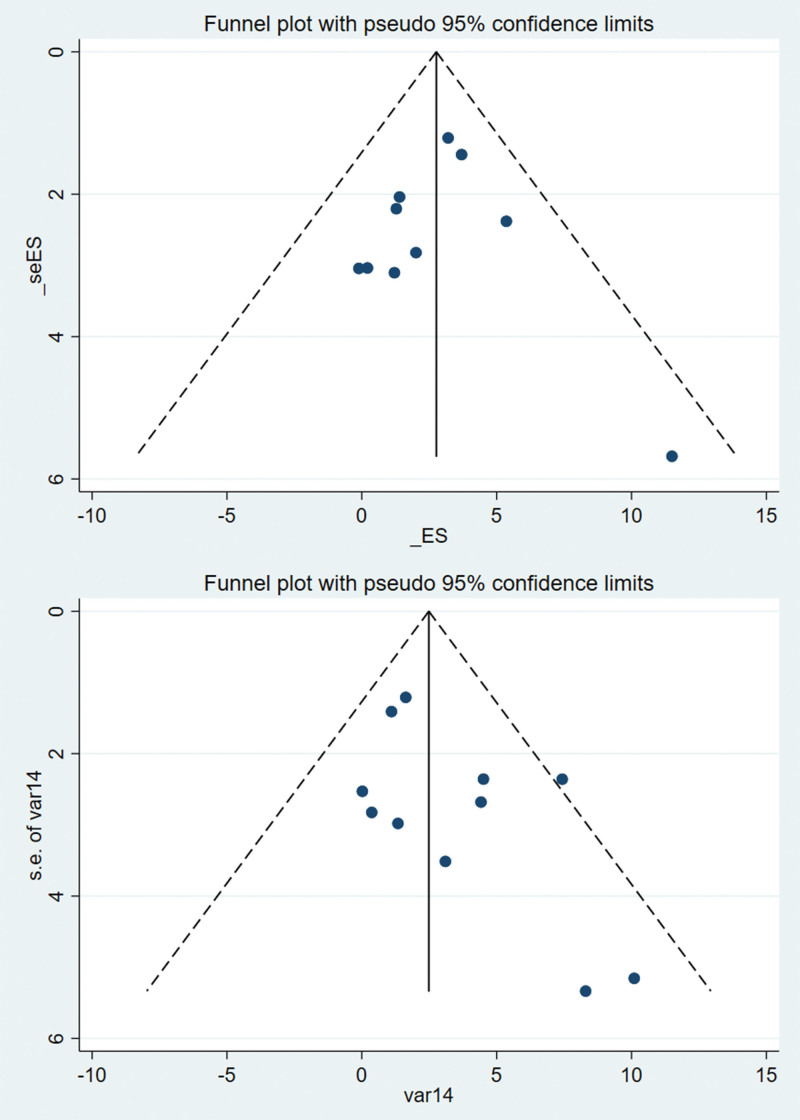
Funnel plot of SF-36. SF-36 = 36-item short-form.

**Figure 11.**
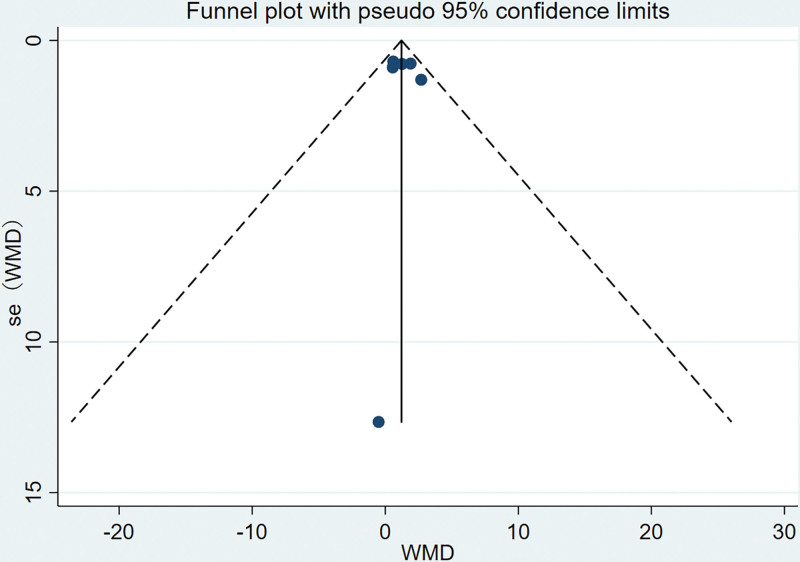
Funnel plot of BBS. BBS = Berg balance scale.

Funnel plots were used to assess publication bias for the WOMAC, SF-36, and BBS. Subsequently, quantitative analysis was performed to evaluate publication bias using Egger’s test. The findings revealed that all *P* values obtained from Egger’s test were greater than 0.05 for WOMAC [pain (p Egger = 0.33), stiffness (p Egger = 0.471), physical function (p Egger = 0.355)], SF-36 [physical score (p Egger = 0.091) and psychological status (p Egger = 0.17)], and BBS (p Egger = 0.75), suggesting the absence of publication bias. Figures 9–11 display the funnel plots for WOMAC, SF-36, and BBS, while the Supplemental Digital Content 10, http://links.lww.com/MD/N485 presents the Egger test plots.

## 4. Discussion

The aim of this study was to investigate the effectiveness of TCE in the treatment of KOA and provide evidence-based medical insights. A comprehensive meta-analysis was conducted, incorporating 20 RCTs involving 1367 patients diagnosed with KOA. The results of the meta-analysis demonstrated that TCE exhibited a significant capacity to alleviate pain, joint stiffness, and other associated symptoms experienced by KOA patients. Furthermore, TCE demonstrated efficacy in improving the psychological well-being and physical functionality of patients, as evidenced by enhancements in their balancing ability, standing up speed, and walking speed. Subgroup analysis, based on the exercise duration, revealed that both long-term habitual TCE, lasting for more than 12 weeks, and short-term TCE, within 12 weeks, were superior to the control group in terms of pain relief, reduction of joint stiffness, and improvement in physical function, as measured by the WOMAC and SF-36 scales. Additionally, in the short term, TCE exhibited a positive impact on psychological scores among KOA patients. However, the analysis of secondary outcomes suggests that long-term TCE (>12 weeks) was more effective in improving the Berg Balance Scale (BBS) score compared to the control group. It is important to note that the limited number of RCTs included in the analysis of secondary outcomes may have influenced this particular finding. The subgroup analysis, based on exercise types, indicated the significant effectiveness of Tai Chi, with only the stiffness subscale of WOMAC showing significant efficacy for Wuqinxi. No statistically significant differences were found for the subgroup analysis of Baduanjin. On the other hand, the scarcity of studies on Yijinjing warrants the need for further RCT results to comprehensively analyze its effectiveness.

Recent research has provided evidence linking KOA, a degenerative and progressive joint disease, with factors such as aging, trauma, obesity, and increased mechanical load.^[68]^ In addition, the inflammation of the knee joint, characterized by pain, swelling, and stiffness, is aggravated by the secretion of cytokines and chemokines from tissues like intra-articular cartilage, meniscus, infrapatellar fat pad, and synovium.^[69]^ Complementary therapy using traditional Chinese medicine(TCM) is commonly employed to treat KOA. This approach promotes the differentiation of bone marrow mesenchymal stem cells and inhibits the release of inflammatory factors and chemokines through various methods such as acupuncture, moxibustion, herbal medicine, Tuina, and traditional Chinese exercises, leading to effective treatment of KOA.^[70]^ KOA is classified under the categories of “bone impediment” and “bi syndrome” in TCM. The primary pathogenesis involves the hindrance of Qi and blood circulation due to a variety of factors.^[71]^ TCM considers Qi as a fundamental concept and invaluable component in maintaining a healthy body. Qi, often referred to as internal energy, plays a pivotal role in regulating bodily functions, disease progression, and the harmonious circulation of vital substances like blood.^[72]^ Previous research has indicated that the utilization of physiotherapy and herbal medicine in TCM has the potential to regulate Qi and blood flow, as well as enhance blood circulation. This approach can effectively strengthen tendons, bones, and joints, thereby alleviating knee pain and ultimately enhancing the overall quality of life for patients with KOA.^[73]^ Such interventions offer promising prospects for both the treatment and prevention of KOA. TCE, as an exercise rooted in traditional Chinese medicine theory, aims to synchronize the actions and consciousness of patients. By doing so, it promotes the alignment of internal and external factors, enhances the functioning of visceral organs, and balances Qi and blood. Additionally, it optimizes the internal movement of Yin and Yang,^[72,74]^ thereby facilitating the alleviation of blood circulation issues and the removal of blood stasis in the treatment of KOA.

An increasing number of studies have been conducted to explore the therapeutic effects of TCE on KOA, given its global popularity. However, the majority of these studies have focused on a single type of TCE,^[75–77]^ and the availability of meta-analyses encompassing multiple types of TCEs has been limited thus far. Given the wide range of TCEs available, it is important to acknowledge this limitation. Our findings demonstrate the efficacy of TCE in alleviating symptoms and improving physical function in patients with KOA. These results align with two prior meta-analyses on the subject.^[42,78]^ However, there are limitations in both studies. The study conducted by Zhang^[78]^ in 2017 had a limited coverage of studies, including only 8 studies with 375 patients involved. It is worth noting that one of the 8 studies did not restrict the participants to those with KOA,^[79]^ which could potentially affect the final results. Li’s study conducted in 2020^[42]^ aimed to evaluate the effectiveness of TCE for KOA by utilizing WOMAC and KOOS indicators, specifically focusing on pain, stiffness, and physical function. It is worth noting that the study conducted subgroup analysis, but it did not provide any information regarding whether the subgroups were a potential source of heterogeneity.

Our meta-analysis offers significant advantages in the following ways: Subgroup analysis has been conducted based on exercise duration, distinguishing between long-term exercise (>12 weeks) and short-term exercise (≤12 weeks), as well as different types of exercise. This comprehensive approach provides valuable guidance for clinicians in formulating scientifically sound traditional Chinese exercise therapies for patients. Our study surpasses previous similar studies in terms of the number of included studies (20) and the number of patients analyzed (1367), thus increasing the reliability and generalizability of our findings. The lengths of follow-up for the included RCTs in our meta-analysis ranged from 8 to 48 weeks, which represents a longer duration compared to previous studies. This longer follow-up period allows for a more comprehensive assessment of the effectiveness of traditional Chinese exercise therapies. However, it is important to acknowledge the following limitations: Due to the nature of exercise therapy as an intervention, it is not possible to completely blind the participants, which may introduce potential bias through psychological suggestion. There exist different versions of TCE, each with its specific details. For instance, Tai Chi can be further categorized into Yang style, Wu style, and Sun style based on the style and form employed. These variations should be taken into account when interpreting the results.^[80]^ However, it is worth noting that the versions of TCE used in the 15 selected RCTs were not entirely consistent. Additionally, the sample sizes of these RCTs were generally small, with less than a third of the studies having a sample size exceeding 50.^[37–39,41,58,66,67]^ Furthermore, it is important to mention that almost half of the 15 RCTs included in this meta-analysis did not provide any information about adverse events that may have occurred during TCE interventions, specifically in studies.^[37,38,41,54,57–59]^ Additionally, one study reported potential adverse effects associated with TCE.^[55]^ Therefore, further research is required to determine the safety of TCE for KOA. Nonetheless, the findings of this meta-analysis have indicated a significant improvement in the overall condition of patients with KOA following TCE intervention.

## 5. Conclusion

This meta-analysis has demonstrated the efficacy of TCE in improving both the symptoms and physical function of KOA patients. Furthermore, both short-term (<12 weeks) and long-term TCE (>12 weeks) have been found to effectively alleviate key symptoms of KOA, such as joint pain, stiffness, and limited mobility, when compared to traditional physical therapy. Among the various forms of TCE, Tai Chi has been widely recognized for its remarkable efficacy. However, it is important to acknowledge that the efficacy of TCE may be overstated due to the generally small sample sizes in the included RCTs, the inability to conduct comprehensive blinding on participants, and the existence of publication bias. In order to establish TCE as a recommended alternative and adjuvant therapy for KOA, further large-scale, long-term interventional studies, conducted at multiple centers with high quality, are required to validate its effectiveness. Thus, additional research is needed to corroborate the benefits of TCE in the treatment of KOA before it can be widely implemented in clinical practice.

## Acknowledgments

We would like to thank the researchers and study participants for their contributions.

## Author contributions

**Conceptualization:** Boyuan Qiu, Weiwei Wang, Gangjian Tang.

**Formal analysis:** Boyuan Qiu, Weiwei Wang, Sheng Chai.

**Funding acquisition:** Zhixue Ou.

**Investigation:** Boyuan Qiu, Weiwei Wang, Gangjian Tang, Sheng Chai, Xuan Zhang, Pengwei Zhou.

**Methodology:** Boyuan Qiu, Gangjian Tang, Xuan Zhang.

**Resources:** Boyuan Qiu, Sheng Chai, Zhixue Ou.

**Supervision:** Xuan Zhang, Pengwei Zhou, Zhixue Ou.

**Writing – original draft:** Boyuan Qiu, Sheng Chai.

**Writing – review & editing:** Weiwei Wang, Gangjian Tang, Xuan Zhang, Pengwei Zhou, Zhixue Ou.

## Supplementary Material


