# Supplementary material for: Long- and short-term effectiveness of traditional Chinese exercises in improving the overall physical capacity of patients with knee osteoarthritis: A systematic review and meta-analysis
Source: Medicine (Baltimore). 2024 Sep 6;103(36):e39520. doi: 10.1097/MD.0000000000039520 (PMC11383713; doi:10.1097/MD.0000000000039520)
Supplement: Supplementary file 1 [file medi-103-e39520-s001.pdf]

Supplemental Digital Content 1. Search Strategy

Database	Search Strategies	Number of Articles
PubMed	(("Osteoarthritis, Knee"[Mesh]) OR (((((((((((((((((Osteoarthritis, Knee[Title/Abstract]) OR (Knee Osteoarthritides[Title/Abstract])) OR (Knee Osteoarthritis[Title/Abstract])) OR (Osteoarthritis of Knee[Title/Abstract])) OR (Osteoarthritis of the Knee[Title/Abstract])) OR (Gonitis[Title/Abstract])) OR (Gonarthritis[Title/Abstract])) OR (Knee Arthritis[Title/Abstract])) OR (arthrosis, knee[Title/Abstract])) OR (femorotibial arthrosis[Title/Abstract])) OR (gonarthrosis[Title/Abstract])) OR (knee arthrosis[Title/Abstract])) OR (knee joint arthrosis[Title/Abstract])) OR (knee joint osteoarthritis[Title/Abstract])) OR (knee osteo-arthritis[Title/Abstract])) OR (knee osteo-arthrosis[Title/Abstract])) OR (knee osteoarthrosis[Title/Abstract])) OR (osteoarthritis, knee[Title/Abstract])) OR (osteoarthrosis, knee[Title/Abstract]))) AND ((("Tai Ji"[Mesh]) OR ("Qigong"[Mesh])) OR (((((((((((((((((((((Qigong[Title/Abstract]) OR (Tai Ji[Title/Abstract])) OR (Qi Gong[Title/Abstract])) OR (Ch'i Kung[Title/Abstract])) OR (Yijinjing[Title/Abstract])) OR (Baduanjin[Title/Abstract])) OR (Wuqinxi[Title/Abstract])) OR (Wu Qin xi[Title/Abstract])) OR (Tai-ji[Title/Abstract])) OR (Tai Chi[Title/Abstract])) OR (Chi, Tai[Title/Abstract])) OR (Tai Ji Quan[Title/Abstract])) OR (Ji Quan, Tai[Title/Abstract])) OR (Quan, Tai Ji[Title/Abstract])) OR (Taiji[Title/Abstract])) OR (Taijiquan[Title/Abstract])) OR (T'ai Chi[Title/Abstract])) OR (Tai Chi Chuan[Title/Abstract])) OR (chi kung[Title/Abstract])) OR (chigung[Title/Abstract])) OR (traditional Chinese exercise[Title/Abstract])) OR (Taiji quan[Title/Abstract])))	131
Web of Science	Osteoarthritis, Knee (Topic) or Knee Osteoarthritides (Topic) or Knee Osteoarthritis (Topic) or Osteoarthritis of Knee (Topic) or Osteoarthritis of the Knee (Topic) or Gonitis (Topic) or Gonarthritis (Topic) or Knee Arthritis (Topic) or knee osteoarthritis (Topic) or arthrosis, knee (Topic) or femorotibial arthrosis (Topic) or arthrosis, knee (Topic) or femorotibial arthrosis (Topic) or gonarthrosis (Topic) or knee arthrosis (Topic) or knee joint arthrosis (Topic) or knee joint osteoarthritis (Topic) or knee osteo-arthritis (Topic) or knee osteo-arthrosis (Topic) or knee osteoarthrosis (Topic) or osteoarthritis, knee (Topic) or osteoarthrosis, knee (Topic) AND Qigong (Topic) or Qi Gong (Topic) or Yijinjing (Topic) or Baduanjin (Topic) or Wuqinxi (Topic) or Wu Qin xi (Topic) or Tai Ji (Topic) or Tai-ji (Topic) or Tai Chi (Topic) or Chi, Tai (Topic) or Tai Ji Quan (Topic) or Ji Quan, Tai (Topic) or Quan, Tai Ji (Topic) or Taiji (Topic) or Taijiquan (Topic) or T'ai Chi (Topic) or Tai Chi Chuan (Topic) or qigong (Topic) or baduanjin (Topic) or wuqinxi (Topic) or chigung (Topic) or traditional Chinese exercise (Topic) or Tai Chi (Topic) or Taiji quan (Topic)	276
Cochrane Library	(Osteoarthritis, Knee)MeSH or ((((((Osteoarthritis, Knee):ti,ab,kw OR (Knee Osteoarthritides):ti,ab,kw OR (Knee Osteoarthritis):ti,ab,kw OR (Osteoarthritis of Knee):ti,ab,kw OR (Osteoarthritis of the Knee):ti,ab,kw) or ((((((Gonitis):ti,ab,kw OR (Gonarthritis):ti,ab,kw OR (Knee Arthritis):ti,ab,kw OR (knee osteoarthritis):ti,ab,kw OR (arthrosis, knee):ti,ab,kw and (Qigong)MeSH) or (Tai Ji)MeSH or (((((((Qigong):ti,ab,kw OR (Qi	150

	Gong):ti,ab,kw OR (Ch'i Kung):ti,ab,kw OR (Yijinjing):ti,ab,kw OR (Baduanjin):ti,ab,kw) or ((((((Wuqinxi):ti,ab,kw OR (Wu Qin xi):ti,ab,kw OR (Tai Ji):ti,ab,kw OR (Tai-ji):ti,ab,kw OR (Tai Chi):ti,ab,kw) or ((((((Chi, Tai):ti,ab,kw OR (Tai Ji Quan):ti,ab,kw OR (Ji Quan, Tai):ti,ab,kw OR (Quan, Tai Ji):ti,ab,kw OR (Taiji):ti,ab,kw) or ((((((Taijiquan):ti,ab,kw OR (T'ai Chi):ti,ab,kw OR (Tai Chi Chuan):ti,ab,kw OR (qigong):ti,ab,kw OR (baduanjin):ti,ab,kw) or ((((((wuqinxi):ti,ab,kw OR (chi kung):ti,ab,kw OR (traditional Chinese exercise):ti,ab,kw OR (Tai Chi):ti,ab,kw OR (Taiji quan):ti,ab,kw)	
EMBASE	(('knee osteoarthritis'/exp) OR ('knee osteoarthritides':ti,ab,kw OR 'osteoarthritis of knee':ti,ab,kw OR 'osteoarthritis of the knee':ti,ab,kw OR gonitis:ti,ab,kw OR gonarthritis:ti,ab,kw OR 'knee arthritis':ti,ab,kw OR 'knee osteoarthritis':ti,ab,kw OR 'arthrosis, knee':ti,ab,kw OR 'femorotibial arthrosis':ti,ab,kw OR gonarthrosis:ti,ab,kw OR 'knee arthrosis':ti,ab,kw OR 'knee joint arthrosis':ti,ab,kw OR 'knee joint osteoarthritis':ti,ab,kw OR 'knee osteo-arthritis':ti,ab,kw OR 'knee osteo-arthrosis':ti,ab,kw OR 'knee osteoarthrosis':ti,ab,kw OR 'osteoarthritis, knee':ti,ab,kw OR 'osteoarthrosis, knee':ti,ab,kw)) AND (((((('qigong'/exp) OR ('baduanjin'/exp) OR ('wuqinxi'/exp) OR ('tai chi'/exp) OR ('qi gong':ti,ab,kw OR yijinjing:ti,ab,kw OR 'wu qin xi':ti,ab,kw OR 'tai ji':ti,ab,kw OR 'chi, tai':ti,ab,kw OR 'tai ji quan':ti,ab,kw OR 'ji quan, tai':ti,ab,kw OR 'quan, tai ji':ti,ab,kw OR taiji:ti,ab,kw OR taijiquan:ti,ab,kw OR 'tai chi chuan':ti,ab,kw OR qigong:ti,ab,kw OR baduanjin:ti,ab,kw OR wuqinxi:ti,ab,kw OR 'chi kung':ti,ab,kw OR chigung:ti,ab,kw OR 'traditional chinese exercise':ti,ab,kw OR 'tai chi':ti,ab,kw OR 'taiji quan':ti,ab,kw))	246
Total Number of Articles		803

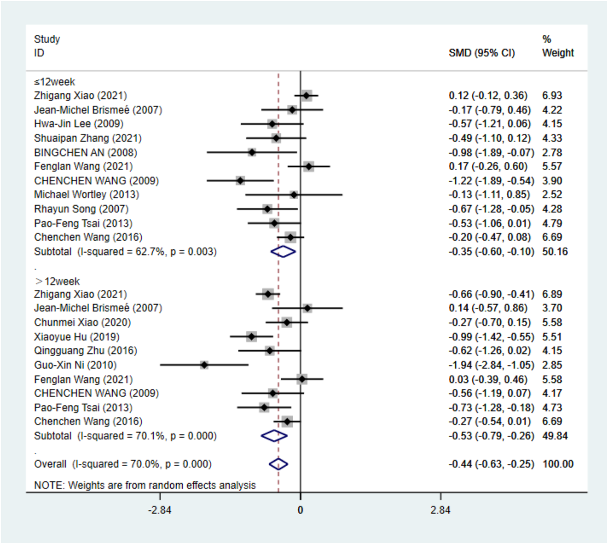

a.

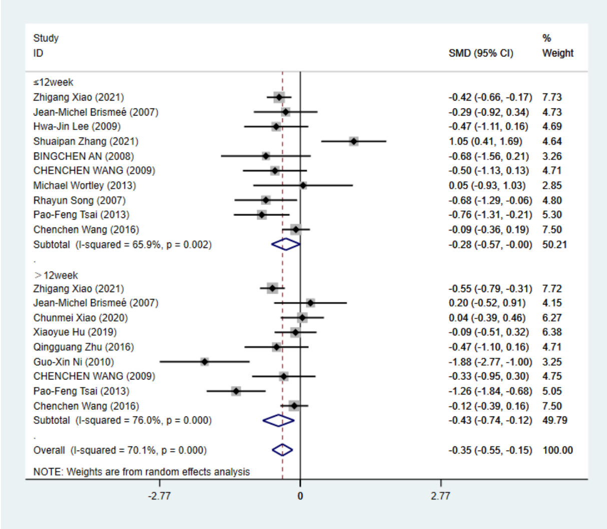

b.

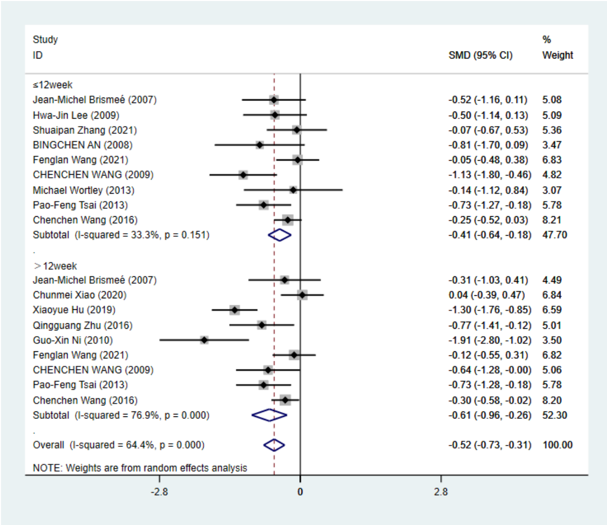

c.

Supplemental Digital Content 2. WOMAC Subgroup Analysis by Follow-up Time (a. pain; b. stiffness; c. function)

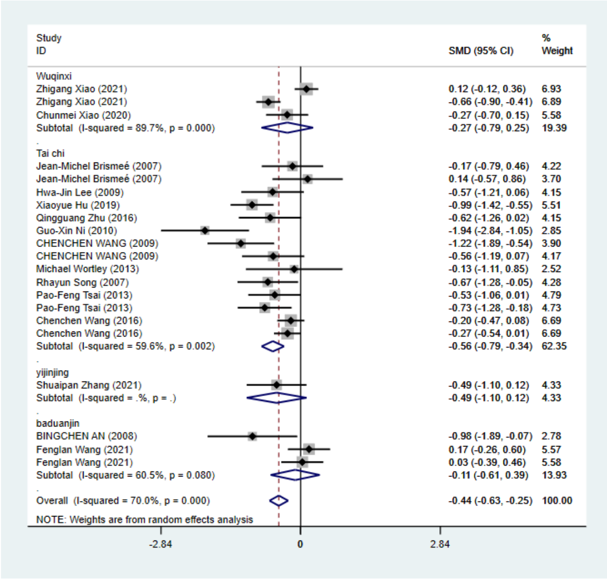

a.

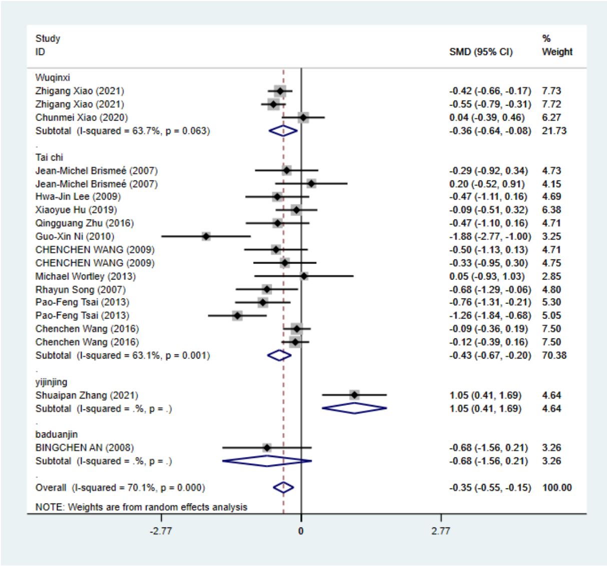

b.

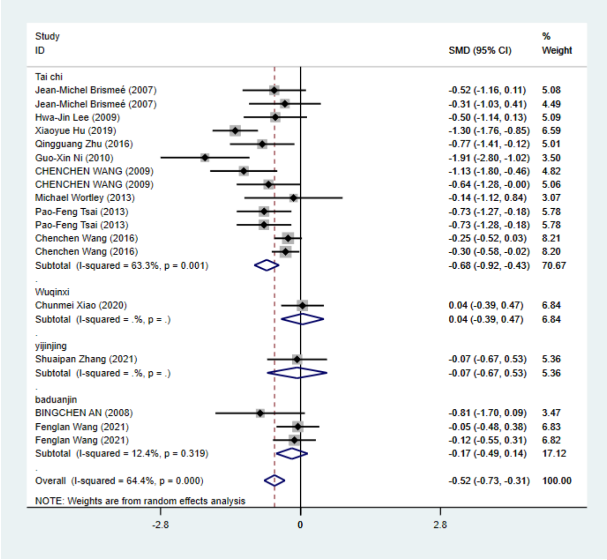

c.

Supplemental Digital Content 3. WOMAC Subgroup Analysis by Exercise Type (a. pain; b. stiffness; c. function)

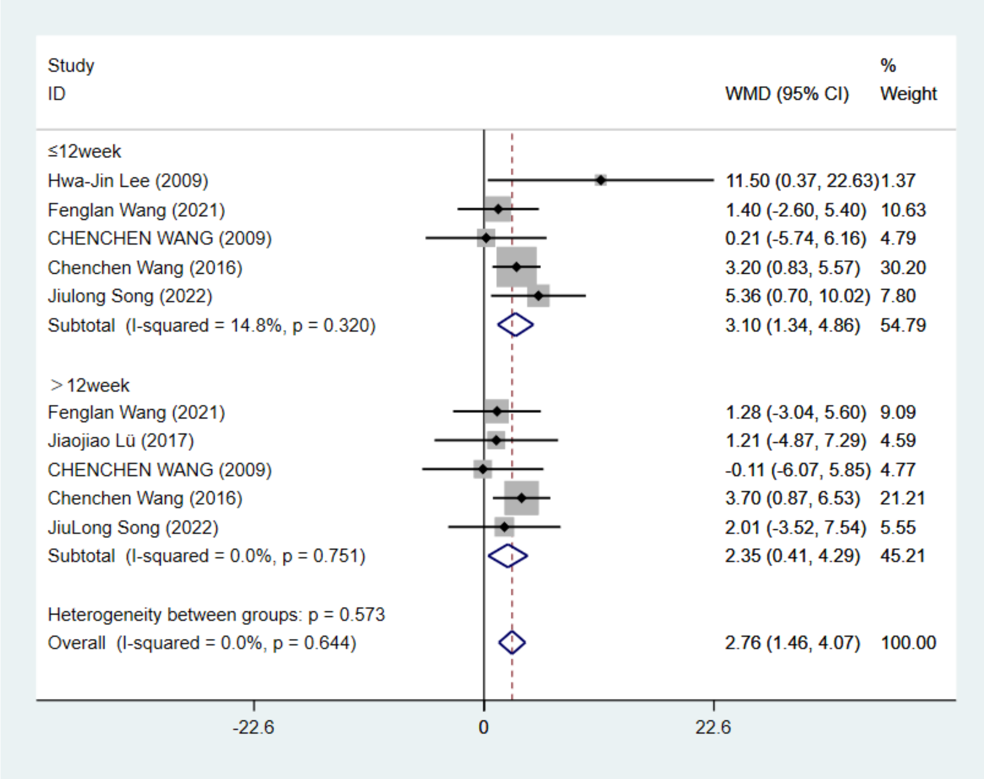

a.

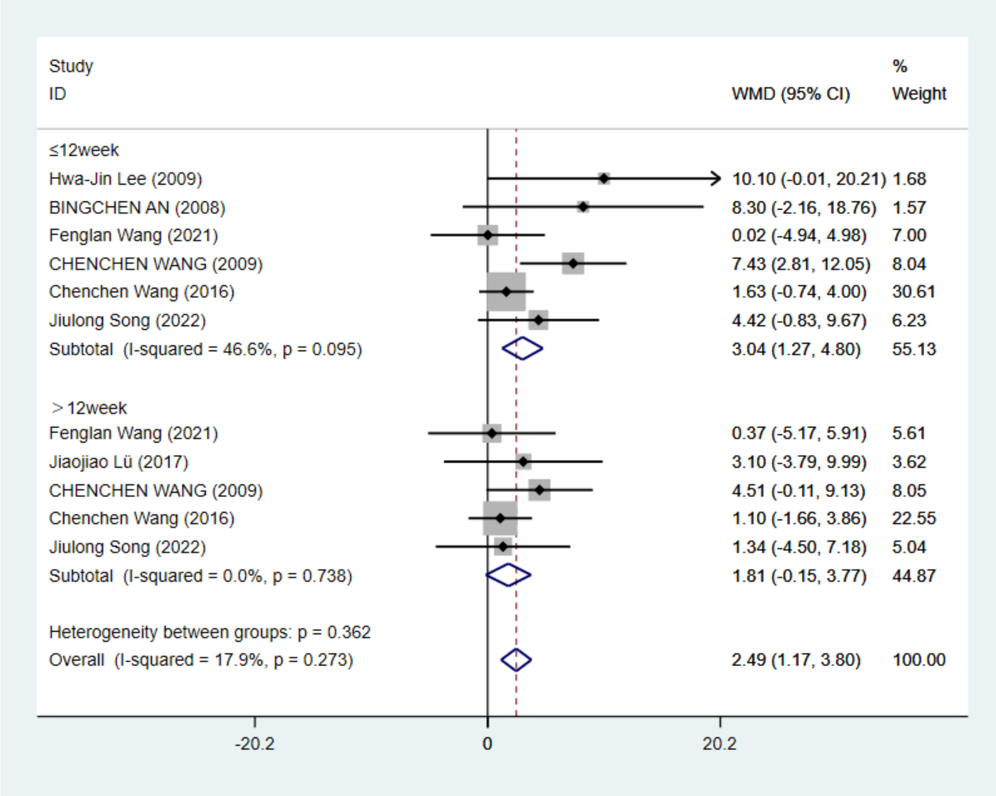

b.

Supplemental Digital Content 4. SF-36 Subgroup Analysis by Follow-up Time (a. physical; b. mental)

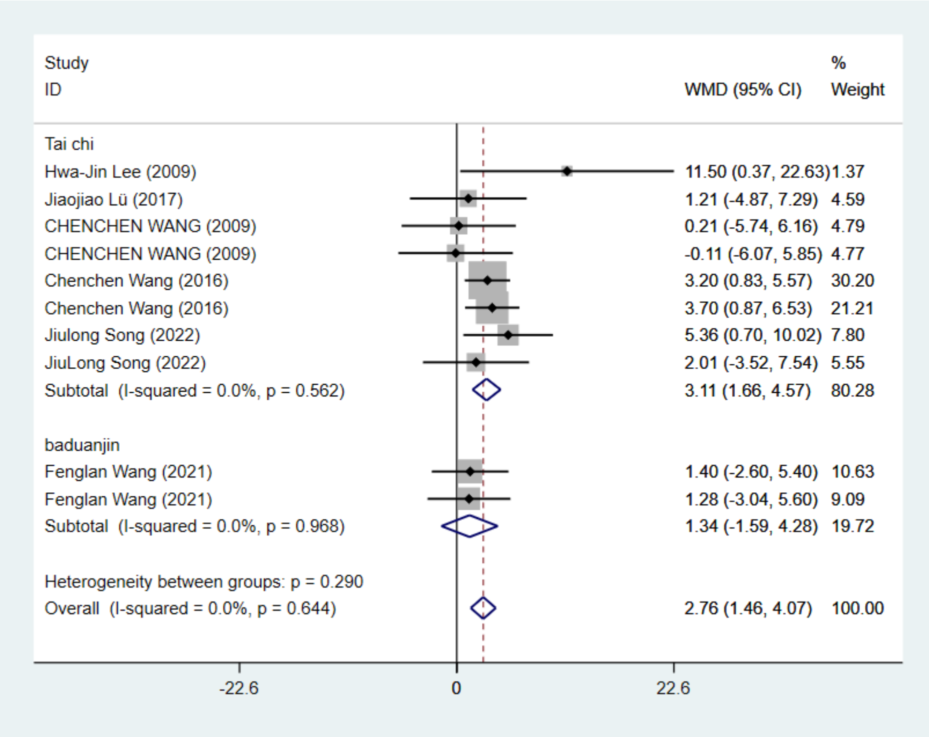

a.

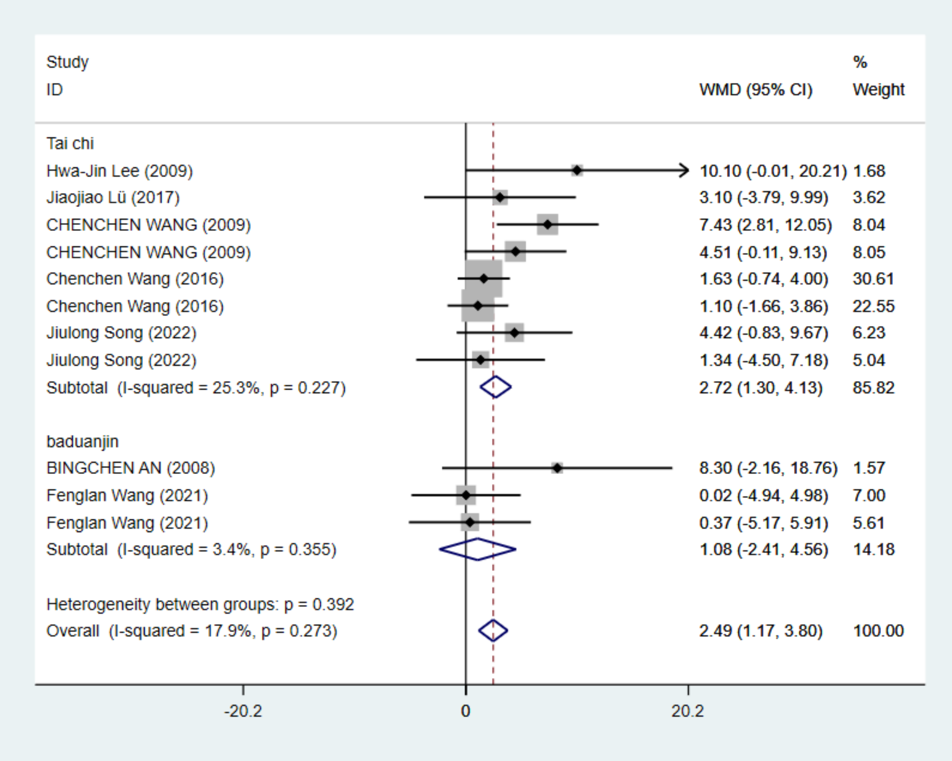

b.

Supplemental Digital Content 5. SF-36 Subgroup Analysis by Exercise Type (a. physical; b. mental)

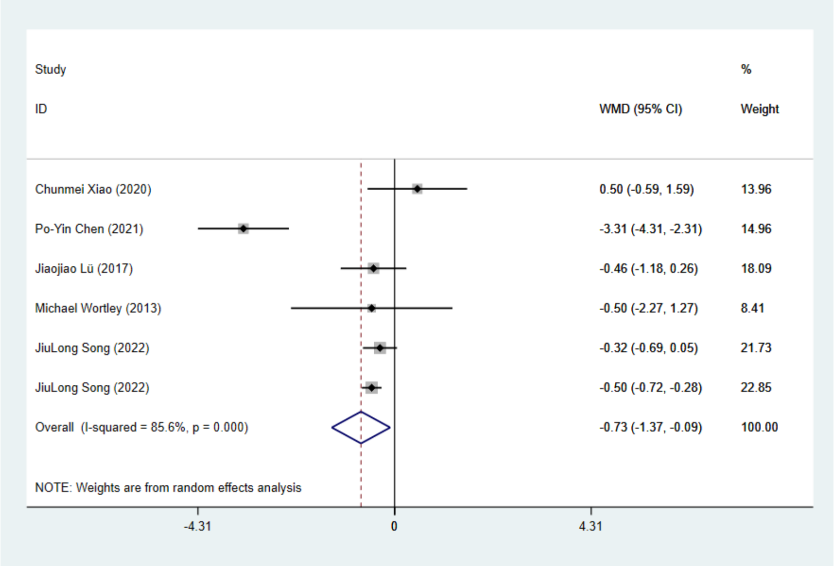

a.

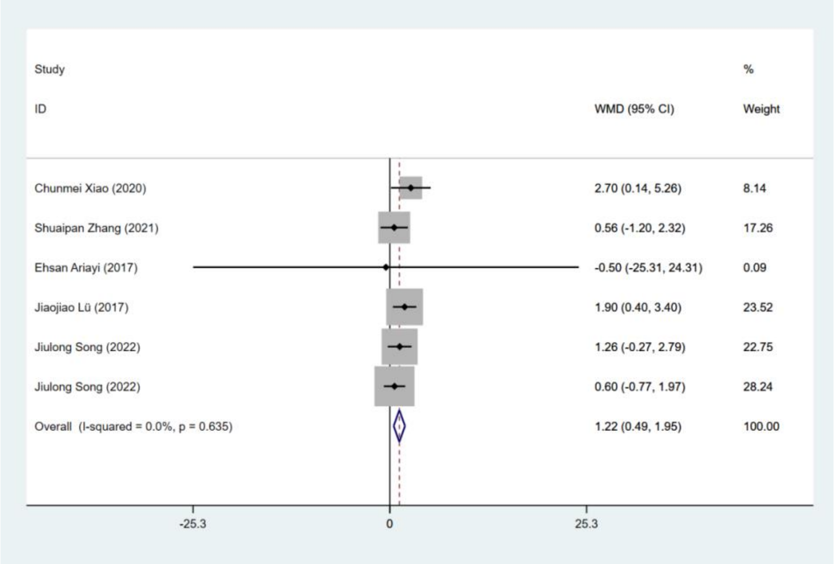

b.

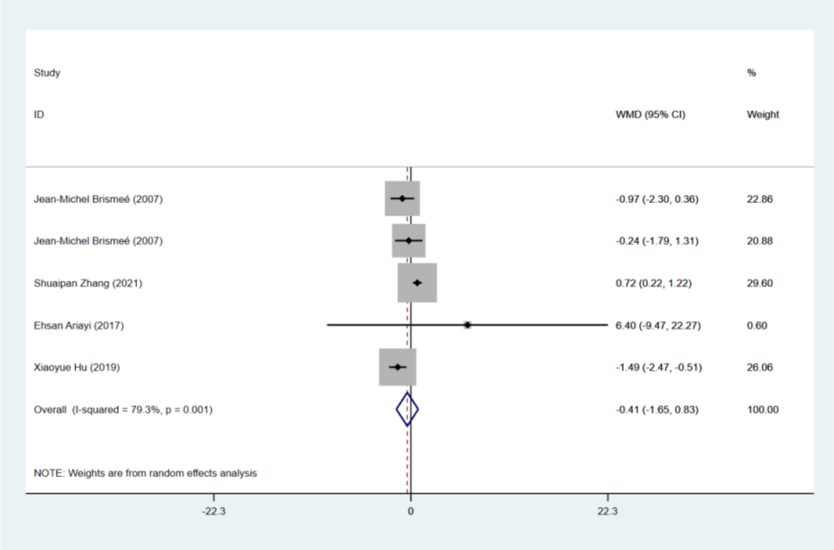

c.

Supplemental Digital Content 6. Meta-analysis of Secondary Outcomes (a. TUG; b. BBS; c. VAS)

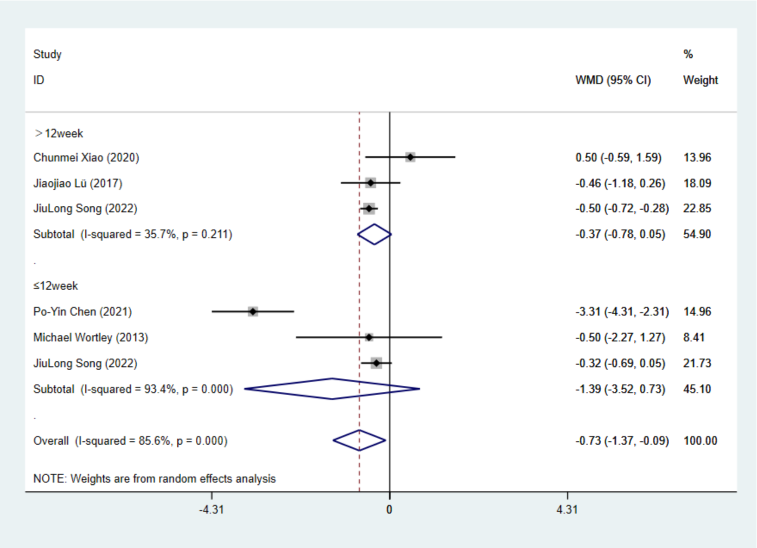

a.

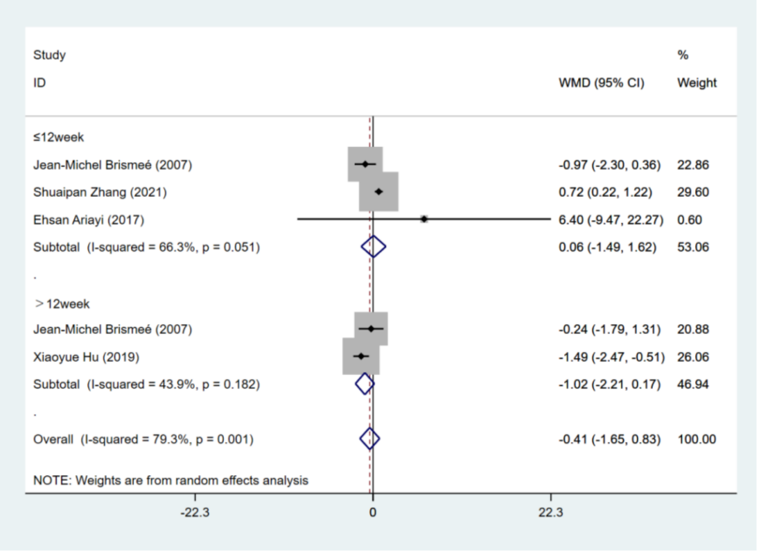

b.

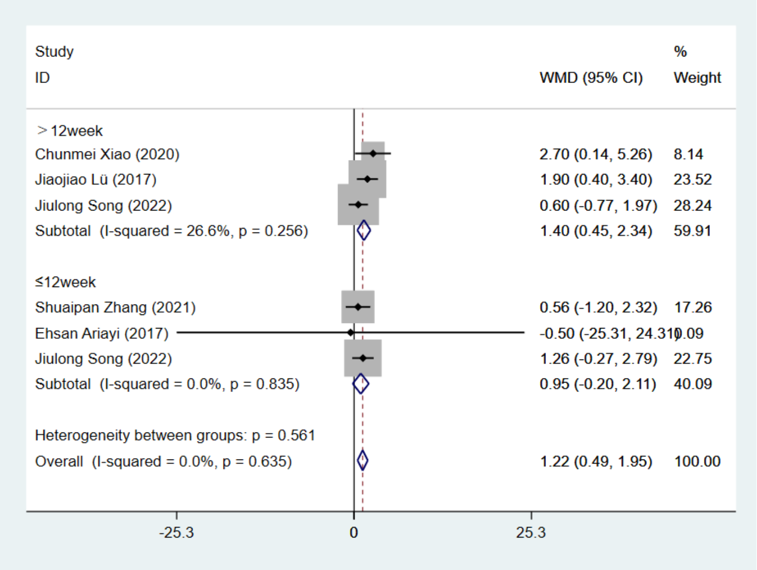

c.

Supplemental Digital Content 7. Subgroup Analysis of Secondary Outcomes by Follow-up Time (a. TUG; b. VAS; c. BBS)

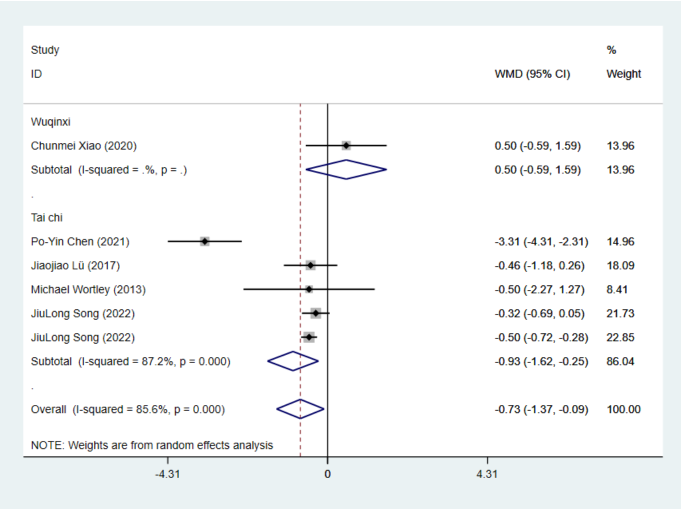

a.

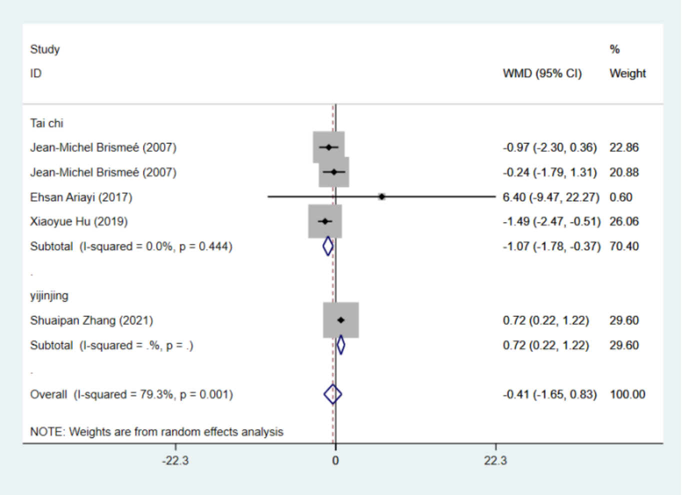

b.

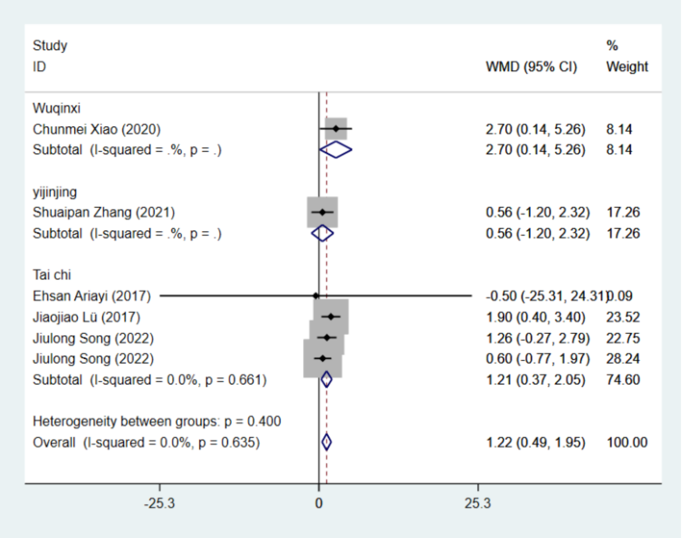

c.

Supplemental Digital Content 8. Subgroup Analysis of Secondary Outcomes by Exercise Type (a. TUG; b. VAS; c. BBS)

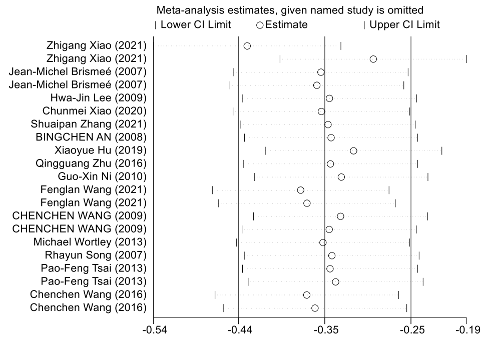

a.

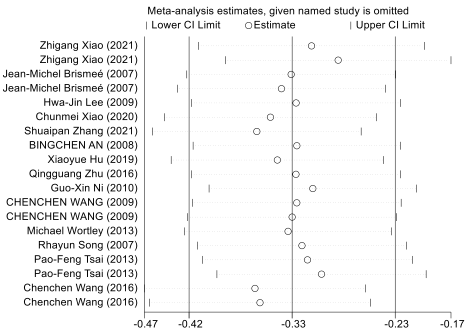

b.

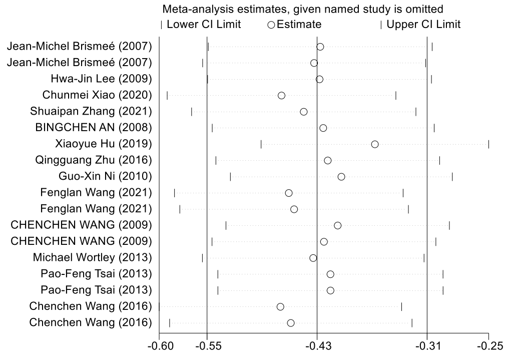

c.

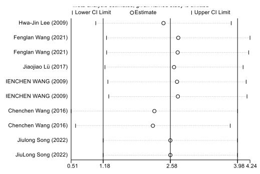

d.

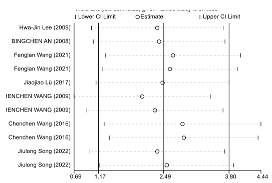

e.

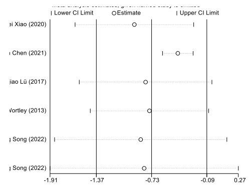

f.

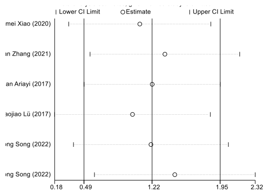

g.

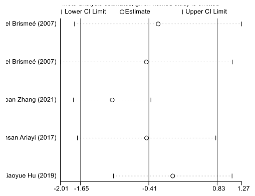

h.

Supplemental Digital Content 9. Sensitivity Analysis

(a.WOMAC- pain;b.WOMAC-stiffness;c.WOMAC-function;d.SF-36-physical;e.SF-36-mental;f.TUG;g.BBS;h.VAS)

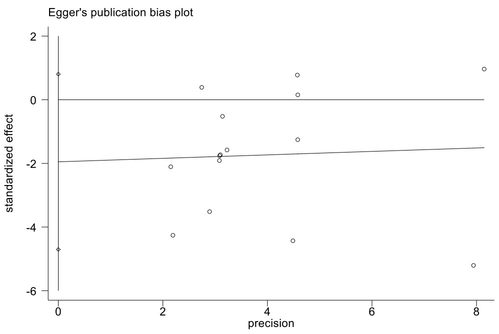

a.

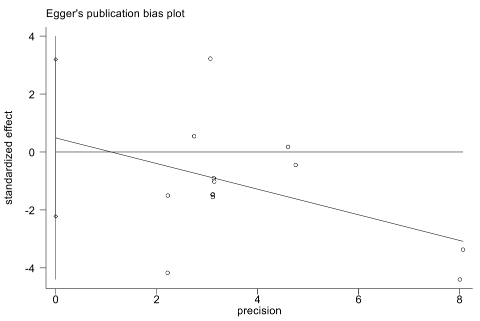

b.

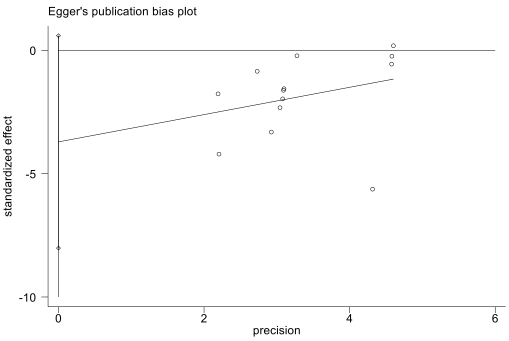

c.

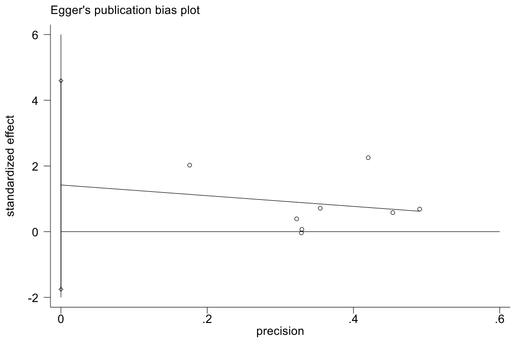

d.

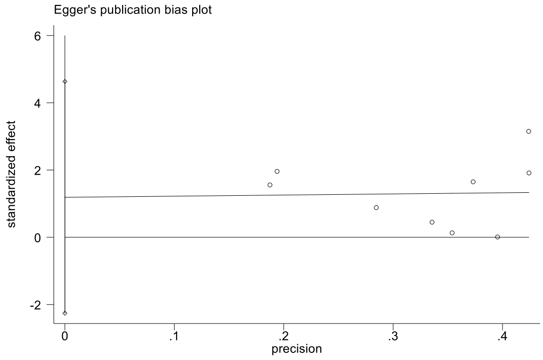

e.

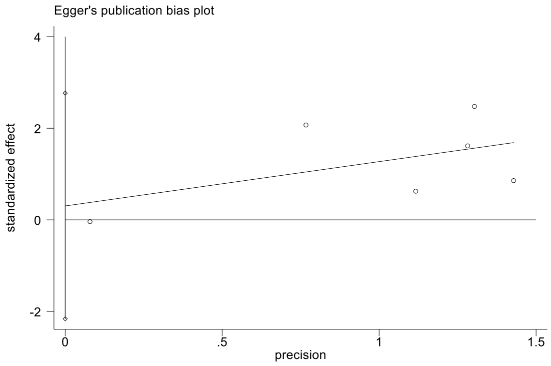

f.

Supplemental Digital Content 10. Egger Test

（a.WOMAC- pain;b.WOMAC-stiffness;c.WOMAC-function;d.SF-36-physical;e.SF-36-mental;f.BBS）
